# Fusion transcriptome landscape in glioblastoma: Incidence and therapeutic implications

**DOI:** 10.1093/noajnl/vdaf238

**Published:** 2025-11-13

**Authors:** Sonikpreet Aulakh, Joanne Xiu, Shawn Kothari, Soma Sengupta, Negar Sadeghipour, Michael Glantz, Manmeet S Ahluwalia, Theodore Nicolaides, Mark R Gilbert

**Affiliations:** Department of Medical Oncology, West Virginia University, Morgantown; CARIS Life Sciences, Phoenix; Northwestern University, Chicago; Departments of Neurology and Neurosurgery, University of North Carolina, Chapel Hill; CARIS Life Sciences, Phoenix; Department of Neurosurgery, Penn State Milton S. Hershey Medical Center, Hershey; Department of Medical Oncology, Miami Cancer Institute, Baptist Health South Florida, Miami; CARIS Life Sciences, Phoenix; National Institutes of Health, Bethesda

**Keywords:** clinical trials, gene fusions, glioblastoma, molecular profiling, targeted therapy

## Abstract

**Background:**

Glioblastoma (GBM) lacks effective therapies for recurrent disease. Unlike cancers with successful fusion-targeted treatments (eg *BCR*-*ABL1* in CML), the incidence and therapeutic potential of gene fusions in GBM remain unclear. We analyzed a large genomic database to define fusion frequency and molecular associations.

**Methods:**

4800 *IDH*-wildtype GBM samples (WHO 2021) underwent NextGen DNA sequencing (592-gene panel/whole exome) and Whole Transcriptome Sequencing for fusions at Caris Life Sciences. Fisher-Exact/Chi-Square tests, adjusted by Benjamini-Hochberg (q < 0.05), assessed significance.

**Results:**

Pathogenic fusions occurred in 428 (8.9%) samples, primarily *FGFR3* (37%, *n* = 159; *FGFR3: TACC3*, *n* = 134), *MET* (21%, *n* = 92), and *EGFR* (20%, *n* = 87). Pathogenic or likely pathogenic fusions included *NTRK2* (*n* = 27), *PDGFRA* (*n* = 23), *ROS1* (*n* = 14), and *BRAF* (*n* = 10). Fusion-positive tumors had higher *MET* (7.5% vs. 0.7%), *FGFR3* (5% vs. 0.2%), *CDK4* (17% vs. 11%), and *MDM2* (12% vs. 7.5%) amplifications, but lower *EGFR* mutations (6.1% vs. 18%), amplifications (6.1% vs. 18%), and *EGFRvIII* (11.9% vs. 22.5%) (all q < 0.05). Median survival was 16.6 months (fusion-positive) vs. 15.5 months (fusion-negative) (*P* = 0.043). Tyrosine kinase inhibitor (TKI)-treated fusion-positive patients (*n* = 37) showed no significant survival benefit (18.4 vs. 16.5 months, *P* = .971).

**Conclusions:**

Approximately 9% of GBMs harbor targetable fusions, with five genes (*FGFR3*, *MET*, *EGFR*, NTRK2, *PDGFRA*) comprising 8%. These findings support multi-arm clinical trials to evaluate targeted therapies, potentially improving outcomes for molecularly defined GBM subgroups.

Key Points

- 8.9% of GBMs have targetable gene fusions.
- *FGFR3*, *MET*, *EGFR* fusions dominate in GBM.
- Fusions linked to unique molecular profiles.

Importance of the Study

This study provides the largest analysis of gene fusions in IDH-wildtype glioblastoma, revealing an 8.9% incidence across 4,800 samples—far exceeding prior smaller reports. Unlike well-studied fusions in cancers like CML or NSCLC, GBM fusions (eg FGFR3, MET, EGFR) remain underexplored, limiting targeted therapy development. Our findings identify a molecularly distinct subgroup of alterations, contrasting with EGFR-driven GBMs, and suggest a modest survival trend. This addresses a critical gap, as recurrent GBM lacks effective treatments. This data can support multi-arm clinical trials to test fusion-targeted therapies, offering a precision oncology framework for GBM. Translationally, this could lead to routine fusion screening and new salvage options, improving outcomes for patients with limited alternatives. Future efforts may refine trial design and drug selection, leveraging existing inhibitors to tackle this devastating disease.

The identification of imatinib as an effective therapy for chronic myeloid leukemia (CML) harboring the *BCR-ABL1* fusion marked a turning point in cancer treatment, highlighting the potential of targeting gene fusions.^1^ These genetic alterations, resulting from chromosomal rearrangements, often produce oncogenic fusion proteins that drive tumor growth, as seen in CML and other malignancies.^2^ In contrast, glioblastoma (GBM), the most aggressive primary brain tumor, remains incurable despite advances in surgery, radiation, and chemotherapy.^3^ Standard therapy with temozolomide and radiation extends survival to approximately 15 months, but recurrent disease lacks effective options.^3^ Molecular profiling has identified frequent alterations in GBM, such as *EGFR* amplification and *TP53* mutations, yet targeting these has yielded limited success.^4^^,^^5^

Gene fusions, while well-established therapeutic targets in cancers like non-small cell lung cancer (NSCLC) with *EML4-ALK*,^6^ are less studied in GBM. Early reports suggest fusions like *FGFR3: TACC3* and *PTPRZ1: MET* occur in GBM, but their incidence and clinical relevance remain uncertain.^7^^,^^8^^,^^9^^,^^10^ This knowledge gap hampers trial design for fusion-targeted therapies, which have transformed outcomes in other cancers.^11^ For instance, *NTRK* inhibitors have shown efficacy across *TRK* fusion-positive tumors, prompting routine screening in some malignancies.^11^ If similar efficacy could be demonstrated in GBM, it might offer salvage therapies for specific patient subsets.

To address this, we analyzed 4800 *IDH*-wildtype GBM samples from a comprehensive genomic database to determine the frequency, spectrum, and molecular correlates of gene fusions. Our goal was to provide data critical for planning prospective trials, potentially identifying targets to improve GBM outcomes.

## Methods

### Patient Population

We analyzed 4800 GBM specimens submitted to Caris Life Sciences (Phoenix, AZ) between 2009 and 2024. Diagnoses adhered to WHO 2021 criteria^12^^,13^, confirmed by Caris pathologists, and all tumors were *IDH1/2*-wildtype. Samples were retrospectively assessed for fusions via Whole Transcriptome Sequencing (WTS). This study was exempt per 45 CFR 46.101(b), with deidentified data.

### Molecular Profiling

DNA mutations were detected using NextGen sequencing (592-gene panel or whole exome sequencing). For fusion detection, formalin-fixed paraffin-embedded samples were microdissected to ≥20% tumor nuclei, mRNA extracted, and reverse-transcribed. Anchored multiplex PCR (ArcherDx FusionPlex Solid Tumor panel) enriched target regions, ­followed by sequencing on the Illumina MiSeq platform.^14^

### Tumor Profiling and Fusion Detection

Gene fusion detection was performed on mRNA isolated from a formalin-fixed paraffin-embedded tumor sample using the Illumina NovaSeq platform (Illumina, Inc., San Diego, CA) and Agilent SureSelect Human All Exon V7 bait panel (Agilent Technologies, ƒ, CA). FFPE specimens underwent pathology review and a minimum of 10% of tumor content in the area for microdissection was required. Qiagen RNA FFPE tissue extraction kit was used, and the RNA ­quality and quantity was determined using the Agilent TapeStation. Biotinylated RNA baits were hybridized to the synthesized and purified cDNA targets and the bait-target complexes were amplified in a post capture PCR reaction. Raw WTS data in FASTQ files was demultiplexed, trimmed, counted, and sequences aligned to human reference genome hg19 (WTS) or hg38 (Hybrid) by Spliced Transcripts Alignment to a Reference (STAR) (RRID: SCR_004463) [Dobin 2013]. Clinically-relevant fusions and/or splice variants were detected using STAR-Fusion. A fusion was called “detected” if the number of junction reads + (3X number of spanning reads) ≥3 (except for *ALK* fusions). An *EML4: ALK* fusion was called “detected” if either 1 junction read or 1 spanning read was present. Non-clinical fusions and/or splice variants were called “detected” if the number of junction reads + (3X number of spanning reads) ≥7. Any results not meeting the criteria above were called “not detected.” Analytical validation of this test demonstrated ≥97% Positive Percent Agreement (PPA), ≥99% Negative Percent Agreement (NPA) and ≥99% Overall Percent Agreement (OPA) with a validated comparator method. All the tests were performed in a CLIA certified laboratory, and the results were provided to treating physicians for clinical use.

For genomic analysis with the NextSeq platform (Illumina), a custom designed SureSelect XT assay was used to enrich 592 whole-gene targets (Agilent Technologies). For NovaSeq analysis (WES), a hybrid pull-down panel of baits designed to enrich for more than 700 clinically relevant genes at high coverage (>500×) and high read-depth was used, along with another panel designed to enrich for an additional >20 000 genes at lower depth (>250×). Genetic variants identified were interpreted by board-certified molecular geneticists and categorized as pathogenic or likely pathogenic according to the American College of Medical Genetics and Genomics (ACMG) standards. Only pathogenic or likely pathogenic variants were included in the analyses.

*PD-L1* expression was evaluated on FFPE slides using the SP142 antibody (Spring Biosciences) and considered positive if staining intensity on tumor cell membranes was ≥2+ and >5% of cells were stained as assessed by a board-certified pathologist.

*MGMT* promoter methylation was evaluated by pyrosequencing. DNA extraction from FFPE was performed for subsequent pyrosequencer-based analysis of 5 CpG sites (CpGs 74-78). All DNA samples underwent a bisulfite treatment and were PCR amplified with primers specific for exon 1 of *MGMT* (GRCh37/hgl9—chr10: 131,265,448-131,265,560). Methylation status of PCR amplified products is determined using the PyroMark system. Samples with ≥7% and <9% methylation are considered to be equivocal or gray zone results.

### Statistical Analysis

Real-world overall survival (rwOS) information was obtained from insurance claims data and calculated from tissue collection to last contact, while time-on-treatment (TOT) was calculated from first to last of treatment time. Hazard ratio (HR) was calculated using the Cox proportional hazards model, and *P* values were calculated using the log-rank test. The survival analysis was restricted to patients without prior TMZ/radiation, so the newly diagnosed patients are enriched in the dataset. The Fisher exact or Chi-Square test was used whenever appropriate to compare fusion rates between groups (R v3.4.1). *P*  < .05 were considered significant. Significance was tested by Fisher-Exact and Chi-Square and adjusted by Benjamini-Hochberg (q < 0.05).

## Results

### Patient Characteristics

Of 4800 IDH-wildtype GBMs, 428 (8.9%) had detectable fusions (Table 1). Median age (62 years) and gender distribution (44% female in fusion-positive, 41% in fusion-negative) showed no significant differences (*P* = .15).

**Table 1.** Patient Characteristics and Treatment Profile (Treatments Include Any Received During Disease Course)

Category	Subcategory	Fusion positive	Fusion positive (%)	Fusion negative	Fusion negative (%)	Total	*P* value
Total		428	8.9	4372	91.1	4800	
Gender	Female	190	44.4	1784	40.8	1974	.15
	Male	238	55.6	2588	59.2	2826	
Age	Median age	62		62		62	ns
	Range	54-71		53-69		53-69	
Race	Asian or Pacific Islander	13	3.0	118	2.7	131	ns
	Black or African American	24	5.6	248	5.7	272	
	Other	18	4.2	241	5.5	259	
	Unknown	92	21.5	1067	24.4	1159	
	White	281	65.7	2698	61.7	2979	
	Hispanic or Latino	31	7.2	300	6.9	331	
	Not Hispanic or Latino	296	69.2	2971	68	3267	
	Unknown	101	23.6	1101	25.2	1202	
Treatment	Radiation	278	65.0	2929	67	3207	ns
	Temozolomide	257	60.0	2678	61.3	2935	
	Bevacizumab	118	27.6	1436	32.8	1554	
	Lomustine	57	13.3	599	13.7	656	
	Fluorouracil	19	4.4	144	3.3	163	
	Carboplatin	11	2.6	150	3.4	161	
	Pembrolizumab	11	2.6	119	2.7	130	
	Nivolumab	7	1.6	34	0.8	41	
	Ipilumumab	0	0.0	13	0.3	13	

### Fusion Incidence

**Figure 1.**
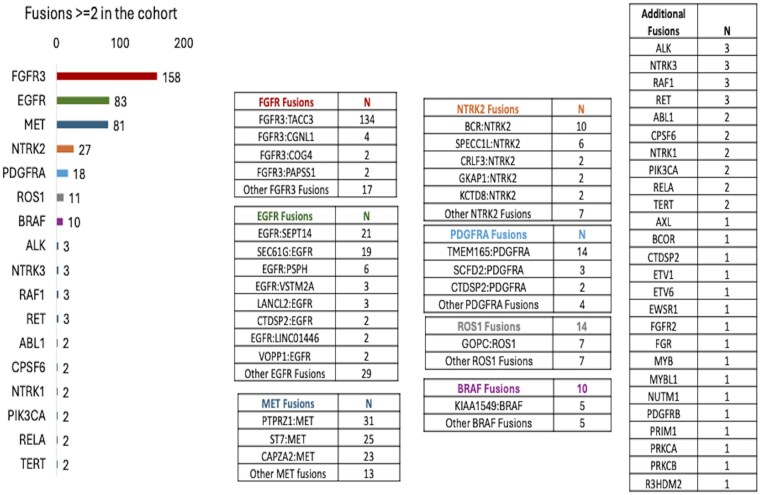
**Prevalence of gene fusions in IDH-Wildtype GBM**. FGFR3, EGFR, and MET were the most common fusions detected.

Pathogenic fusions were identified in 428 samples (8.9%). The most frequent were *FGFR3* (*n* = 159, 37%; *FGFR3: TACC3*, *n* = 134), *MET* (*n* = 92, 21%; *PTPRZ1: MET*, *n* = 31; *ST7: MET*, *n* = 25; *CAPZA2: MET*, *n* = 23), and *EGFR* (*n* = 87, 20%; *EGFR: SEPT14*, *n* = 21; *SEC61G: EGFR*, *n* = 19) (Figure 1). Other fusions included *NTRK2* (*n* = 27), *PDGFRA* (*n* = 23), *ROS1* (*n* = 14), *BRAF* (*n* = 10), *ALK* (*n* = 3), *NTRK3* (*n* = 3), *RAF1* (*n* = 3), *RET* (*n* = 3), and *NTRK1* (*n* = 2). Five genes (*FGFR3*, *MET*, *EGFR*, *NTRK2*, *PDGFRA*) accounted for 8% of samples. Supplementary Table S1 presents a comprehensive dataset of genomic rearrangements leading to fusion transcripts across 428 cases, detailing the involved genes, exon junctions, frame status, and chromosomal locations. The table highlights frequent fusions such as *FGFR3: TACC3* and *EGFR: SEPT14*, with varying read support and breakpoint positions, indicating diverse molecular mechanisms. These findings provide valuable insights into the genetic alterations driving potential therapeutic targets in GBM.

### Molecular Correlates

**Figure 2.**
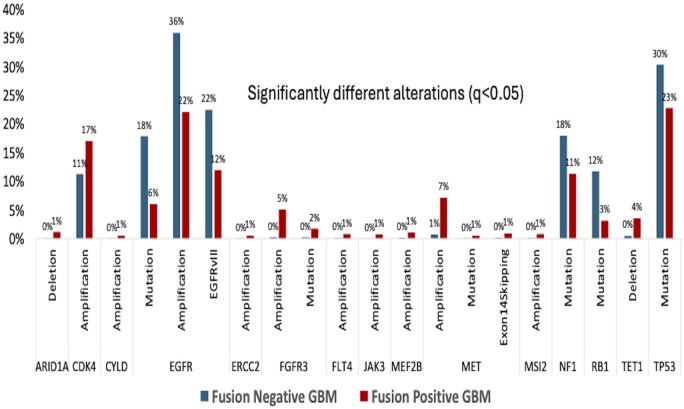
Differences in mutation and amplification incidence between fusion-positive and fusion-negative GBMs.

**Figure 3.**
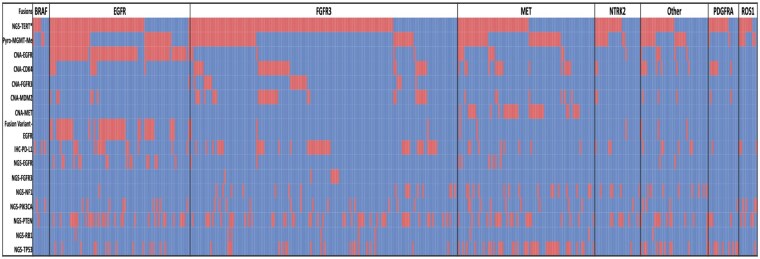
**Oncoprint of Fusion-positive GBMs showing co-occurring alterations (eg EGFR amplification 92%, EGFRvIII 51%, MET amplification 40%)**.

Fusion-positive tumors exhibited distinct molecular profiles (Figure 2). They had higher rates of *MET* amplification (7.5% vs. 0.7%), *FGFR3* amplification (5% vs. 0.2%), *CDK4* amplification (17% vs. 11%), and *MDM2* amplification (12% vs. 7.5%), but lower *EGFR* mutations (6.1% vs. 18%), *EGFR* amplifications (6.1% vs. 18%), *EGFRvIII* mutations (11.9% vs. 22.5%),*RB1* mutations (2.9% vs. 11.5%), and *TP53* mutations (22% vs. 30%) (all q < 0.05). Consistent with *IDH*-wt GBM, ∼60% exhibited +7/-10 signature, that was not significantly different between the Fusion-positive vs. Fusion-negative. Within fusion-positive tumors, *EGFR* fusions co-occurred with *EGFR* amplification (92%) and *EGFRvIII* (51%), while *MET* fusions co-occurred with *MET* amplification (40%) (Figure 3).

### Survival Outcomes

**Figure 4.**
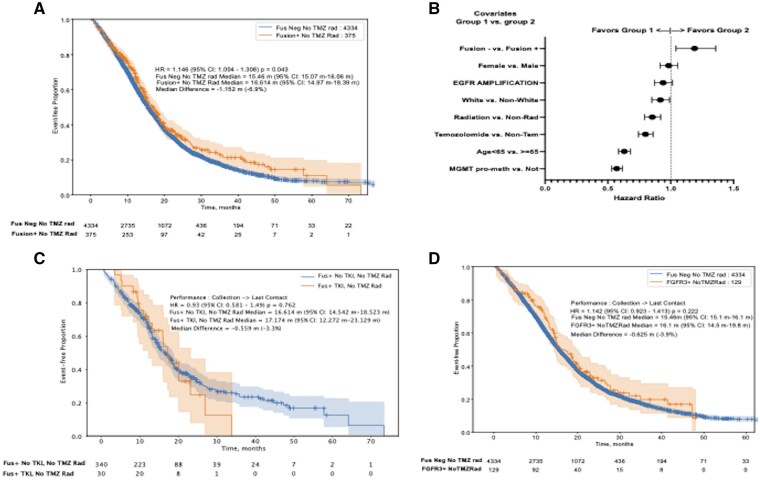
**Survival correlations**. (A) Overall survival (OS) of fusion-positive vs. fusion-negative GBMs. (B) Multivariate analysis of survival factors. (C) OS in TKI-treated vs. untreated fusion-positive patients. (D) OS in FGFR3-positive vs. fusion-negative GBMs.

Median survival was 16.6months (95% CI: 15.0-18.4) for fusion-positive patients (*n* = 375) vs. 15.5 months (95% CI: 15.1-16.1) for fusion-negative patients (*n* = 4334) (*P* = .043, HR = 1.146, 95% CI: 1.004-1.308) (Figure 4A). Multivariate analysis adjusting for age, gender, race, treatment, and biomarkers suggested a favorable trend for fusion-positive status (Figure 4B). Among fusion-positive patients, *TKI*-treated cases (*n* = 30) had a median survival of 17.1 months (95% CI: 12.2-23.1) vs. 16.6 months (95% CI: 14.5-18.5) for untreated cases (*P* = .762, HR = 0.93, 95% CI: 0.581-1.49) (Figure 4C). *FGFR*-positive patients (*n* = 128) showed no survival difference vs. fusion-negative patients (16.1 vs. 15.2 months, *P* = .51, HR = 1.074, 95% CI: 0.868-1.328) (Figure 4D). *FGFR* inhibitor-treated patients (*n* = 18) had no significant survival benefit (*P* = .838).

### Targeted Therapies

Among 128 *FGFR* fusion patients, 18 received *FGFR* inhibitors (eg Erdafitinib, *n* = 5; Infigratinib, *n* = 4), with a median survival of 703 days (range: 252-818) with a treatment duration of 145 days (range: 22-171). Of 65 *EGFR* fusion patients, two received inhibitors (Imatinib, Osimertinib). *NTRK* fusion patients (*n* = 24) received Entrectinib (*n* = 3) or Larotrectinib (*n* = 7), with a median survival of 490 days (range: 217-1029) and treatment duration of 53 days (range: 1-223). *PDGFRA* (*n* = 20) and *ALK* (*n* = 3) fusion patients had limited treatment data (eg Regorafenib, *n* = 1; Alectinib, *n* = 1). Supplementary Table S2 outlines the clinical characteristics of 37 patients treated with tyrosine kinase inhibitors (TKIs), including fusion gene details, TKI regimens, and survival outcomes, with a focus on *FGFR3*, *MET*, and *NTRK2* fusions. The table provides data on overall survival, age, gender, race, and ethnicity, alongside treatment specifics such as post-TMZ administration and survival status (censored or event). These findings highlight the variability in treatment responses and survival across different genetic fusions and patient demographics. Most TKIs (80%) given at recurrence; median TOT 53-145 days. The ∼35% non-receipt rate of RT/TMZ is consistent with real-world patterns observed in elderly patients or those with poor performance status.

## Discussion

This study provides the largest analysis to date of gene fusions in *IDH*-wildtype GBM, identifying an 8.9% incidence across 4800 samples. *FGFR3* (37%), *MET* (21%), and *EGFR* (20%) fusions predominated, with five genes (*FGFR3*, *MET*, *EGFR*, *NTRK2*, *PDGFRA*) comprising 8% of cases. These findings align with smaller studies reporting *FGFR3: TACC3* and *PTPRZ1: MET* in GBM,^7^^,^^8^ but our scale reveals a broader fusion landscape, including rare events (eg *NTRK*, *ROS1*). This contrasts with NSCLC, where *EML4*-*ALK* (5%-7%) drives targeted therapy success,^6^ or *TRK* fusion-positive cancers, where *NTRK* inhibitors achieve high response rates.^11^ In GBM, the lower incidence and heterogeneity of fusions pose challenges for therapeutic development.

Fusion-positive GBMs exhibited distinct molecular profiles, with higher *MET*, *FGFR3*, *CDK4*, and *MDM2* amplifications but reduced *EGFR* alterations. This suggests fusions may define a biologically unique subset, potentially less reliant on *EGFR*-driven pathways, a hallmark of GBM.^4^ Co-occurrence of *EGFR* fusions with *EGFR* amplification (92%) and *EGFRvIII* (51%) mirrors findings in NSCLC, where *ALK* fusions co-occur with *EGFR* mutations.^15^ Similarly, *MET* fusion with *MET* amplification (40%) parallels *MET*-driven cancers.^16^ These patterns indicate fusions may amplify oncogenic signaling, offering multiple therapeutic targets within a tumor.

Survival analysis showed significant difference between fusion-positive and -negative patients.^17^ A favorable trend in multivariate analysis suggests fusions might confer prognostic benefits, possibly masked by heterogeneous treatments or sample size. TKI-treated fusion-positive patients showed no clear survival advantage (18.4 vs. 16.5 months), unlike NSCLC, where *ALK* inhibitors extend survival.^18^ This may reflect suboptimal agent selection (eg Imatinib for *EGFR* fusions vs. Osimertinib), inadequate dosing, or blood-brain barrier penetration issues, a known challenge in GBM.^19^ Additionally, fusion loss in recurrence, as observed in longitudinal GBM studies, may contribute to TKI resistance, emphasizing need for re-biopsy. *FGFR* inhibitor outcomes were similarly inconclusive, possibly due to limited numbers (*n* = 18) or resistance mechanisms, as seen in *FGFR*-driven cancers.^12^^,20^^,21^ Notable cases include, for example, an NTRK fusion-positive patient treated with larotrectinib, achieving a time on treatment of 223 days.

The 8.9% fusion incidence supports the feasibility of clinical trials, but the low frequency of individual fusions (eg *FGFR3: TACC3*, 3%) complicates traditional designs. An “umbrella” trial targeting the five most common fusions could address this, randomizing patients to matched therapies (eg *FGFR* inhibitors for *FGFR3* fusions, *MET* inhibitors for *MET* fusions). With a 92% screen failure rate (fusion-negative cases), prospective screening is impractical without patient registries or cooperative networks like NRG Oncology or the Alliance Oncology Cooperative Group. A hybrid trial design, integrating local care with centralized molecular testing, could reduce costs and improve access, particularly for rural patients.^22^

Precedents exist in other cancers. The NCI-MATCH trial tested targeted therapies across tumor types, identifying responders despite low mutation frequencies.^23^ Similarly, basket trials for *NTRK* fusions demonstrated efficacy across histologies.^9^ Adapting these models to GBM could accelerate drug development, leveraging existing agents (eg Larotrectinib, Crizotinib) while testing novel compounds. However, challenges remain, including standardizing fusion detection (WTS vs. targeted panels), ensuring trial accrual, and addressing resistance, as seen with *EGFR* inhibitors in GBM.^19^

Limitations of this study include the retrospective design, variable treatment data, and lack of progression-free survival metrics. The exact proportion of newly diagnosed to recurrent tumors is not available due to the real-world nature of the study. We focused on pathogenic fusions involving known oncogenes (eg kinases); total fusion burden may be higher if including all events. While orthogonal validation (eg FISH or RT-PCR) was not performed on all cases due to the retrospective nature and limited tissue, the assay’s analytical validation supports reliable detection in FFPE samples with ≥20% tumor content. RNA-based subtyping was not available in this study, as the original microarray-based classification algorithm was not immediately adaptable to the WTS platform. However, the molecular correlates in our study offers important insights on potential enrichment of a subset of proneural subtype for fusion-positive tumors (as suggested by *CDK4*, *FGFR3*, *MET* amplifications) and depletion of classical subtype (as suggested by lower alteration rates in *EGFR*).^21^^,24^ Our future research should be conducted with this approach in mind. Treatment data are limited by small sample size and heterogeneity; progression-free survival (PFS) data are not available. Overall survival (OS) trends are exploratory. Prospective validation is needed to confirm fusion incidence and therapeutic efficacy. Nonetheless, this study establishes a foundation for precision oncology in GBM, identifying a molecularly defined subgroup that could benefit from targeted approaches. Future efforts should integrate multi-omics data (eg proteomics) to elucidate fusion protein function and resistance pathways, enhancing trial design and patient selection.

## Conclusions

Approximately 9% of *IDH*-wildtype GBMs harbor potentially targetable gene fusions, with *FGFR3*, *MET*, *EGFR*, *NTRK2*, and *PDGFRA* comprising 8%. These findings highlight a molecularly distinct subgroup and support the feasibility of multi-arm clinical trials to test targeted therapies. While survival benefits remain unproven, possibly due to limited sample sizes or suboptimal treatments, the data suggest a path forward via innovative trial designs, such as umbrella studies with registries and cooperative networks. Successful implementation could establish new treatment options for GBM and a paradigm for targeting rare molecular alterations in cancer.

## Supplementary Material

vdaf238_Supplementary_Data

## Data Availability

The deidentified sequencing data are owned by Caris Life Sciences. Raw FASTQ files cannot be publicly deposited due to privacy concerns, but summary data (eg fusion lists in Supplementary Tables S1 and S2) are provided. This complies with institutional policies on proprietary clinical data while enabling verification. Data will be made available upon reasonable requests with the permission of Caris Life Sciences. Qualified researchers may contact the corresponding author with their request.
