# Supplementary material for: Fusion transcriptome landscape in glioblastoma: Incidence and therapeutic implications
Source: Neurooncol Adv. 2025 Nov 13;8(1):vdaf238. doi: 10.1093/noajnl/vdaf238 (PMC12831933; doi:10.1093/noajnl/vdaf238)
Supplement: vdaf238_Supplementary_Data [file vdaf238_supplementary_data.zip › Supplementary table 1.docx]

**Supplementary table 1: Genomic rearrangements leading to fusion transcripts**

| Case Number | test | value |
| --- | --- | --- |
| 1 | FGFR3 | FGFR3:TACC3\|exon 17:exon 11\|InFrame\|7\|23\|chr4:1808661:+/chr4:1741429:+ |
| 2 | EGFR | AKAP9:EGFR\|exon 22:exon 18\|InFrame\|4\|0\|chr7:91682272:+/chr7:55241614:+ |
| 3 | FGFR3 | FGFR3:TACC3\|exon 17:exon 10\|InFrame\|15\|0\|chr4:1808661:+/chr4:1739325:+ |
| 4 | FGFR3 | FGFR3:TACC3\|exon 17:exon 8\|InFrame\|399\|9\|chr4:1808661:+/chr4:1737458:+ |
| 5 | ETV6 | ETNK1:ETV6\|exon 1:exon 2\|InFrame\|9\|1\|chr12:22778520:+/chr12:11905384:+ |
| 6 | MET | TNRC6B:MET\|exon 1:exon 2\|NotInFrame\|24\|2\|chr22:40574144:+/chr7:116339125:+ |
| 7 | MET | PTPRZ1:MET\|exon 1:exon 2\|NotInFrame\|20\|0\|chr7:121513611:+/chr7:116339125:+ |
| 8 | ABL1 | BCR:ABL1\|exon 1:exon 2\|InFrame\|4\|0\|chr22:23524426:+/chr9:133729451:+ |
| 9 | EGFR | SEC61G:EGFR\|exon 1:exon 5\|NotInFrame\|582\|0\|chr7:54826851:-/chr7:55218987:+ |
| 10 | MET | CAPZA2:MET\|exon 2:exon 2\|NotInFrame\|5\|0\|chr7:116528244:+/chr7:116339125:+ |
| 11 | MET | PRKAR2A:MET\|exon 9:exon 15\|InFrame\|10\|0\|chr3:48789609:-/chr7:116414935:+ |
| 12 | EGFR | ELMO1:EGFR\|exon 1:exon 2\|NotInFrame\|3\|0\|chr7:37488278:-/chr7:55209979:+ |
| 13 | EGFR | ABCA13:EGFR\|exon 15:exon 2\|InFrame\|36\|4\|chr7:48288948:+/chr7:55209979:+ |
| 14 | BRAF | PTPRJ:BRAF\|exon 18:exon 9\|InFrame\|30\|1\|chr11:48171735:+/chr7:140487384:- |
| 15 | NTRK2 | BCR:NTRK2\|exon 1:exon 16\|InFrame\|59\|7\|chr22:23524426:+/chr9:87482158:+ |
| 16 | MET | ST7:MET\|exon 5:exon 2\|NotInFrame\|24\|3\|chr7:116770660:+/chr7:116339125:+ |
| 17 | EGFR | EGFR:SEPT14\|exon 25:exon 7\|InFrame\|700\|55\|chr7:55269048:+/chr7:55886916:- |
| 18 | MET | FAM133B:MET\|exon 1:exon 3\|InFrame\|5\|0\|chr7:92219582:-/chr7:116371722:+ |
| 19 | FGFR3 | FGFR3:TACC3\|intron 17:exon 6\|NotInFrame\|156\|0\|chr4:1808694:+/chr4:1732965:+ |
| 20 | NTRK2 | SPECC1L:NTRK2\|exon 5:exon 13\|InFrame\|202\|5\|chr22:24720395:+/chr9:87359888:+ |
| 21 | MET | PTPRZ1:MET\|exon 1:exon 2\|NotInFrame\|4\|0\|chr7:121513611:+/chr7:116339125:+ |
| 22 | EGFR | EGFR:VSTM2A\|exon 22:exon 5\|NotInFrame\|430\|14\|chr7:55260534:+/chr7:54636824:+ |
| 23 | FGFR3 | FGFR3:TACC3\|exon 17:exon 11\|InFrame\|107\|1\|chr4:1808661:+/chr4:1741429:+ |
| 24 | EGFR | EGFR:SEPT14\|exon 24:exon 10\|InFrame\|7\|0\|chr7:55268106:+/chr7:55863785:- |
| 25 | FGFR3 | FGFR3:TACC3\|exon 17:exon 10\|InFrame\|13\|4\|chr4:1808661:+/chr4:1739325:+ |
| 26 | FGFR3 | FGFR3:TACC3\|exon 17:exon 10\|InFrame\|10\|2\|chr4:1808661:+/chr4:1739325:+ |
| 27 | FGFR3 | FGFR3:TACC3\|exon 17:exon 10\|InFrame\|264\|5\|chr4:1808661:+/chr4:1739325:+ |
| 28 | FGFR3 | FGFR3:TACC3\|exon 17:exon 10\|InFrame\|4\|0\|chr4:1808661:+/chr4:1739325:+ |
| 29 | MET | PTPRZ1:MET\|exon 1:exon 2\|NotInFrame\|10\|62\|chr7:121513611:+/chr7:116339125:+ |
| 30 | FGFR3 | FGFR3:TACC3\|exon 17:exon 14\|InFrame\|2\|1\|chr4:1808661:+/chr4:1746245:+ |
| 31 | EGFR | LANCL2:EGFR\|exon 5:exon 7\|InFrame\|1\|1\|chr7:55469013:+/chr7:55221704:+ |
| 32 | MET | LHFPL3:MET\|exon 2:exon 2\|NotInFrame\|3\|0\|chr7:104377358:+/chr7:116339125:+ |
| 33 | EGFR | EGFR:HOPX\|exon 24:exon 2\|InFrame\|26\|0\|chr7:55268106:+/chr4:57522178:- |
| 34 | EGFR | PLD5:EGFR\|exon 2:exon 17\|InFrame\|36\|0\|chr1:242511408:-/chr7:55240676:+ |
| 35 | FGFR3 | FGFR3:PAPSS1\|exon 17:exon 6\|InFrame\|36\|1\|chr4:1808661:+/chr4:108581228:- |
| 36 | FGFR3 | FGFR3:TACC3\|exon 17:exon 11\|InFrame\|938\|14\|chr4:1808661:+/chr4:1741429:+ |
| 37 | MET | CAPZA2:MET\|exon 2:exon 2\|NotInFrame\|36\|0\|chr7:116528244:+/chr7:116339125:+ |
| 38 | FGFR3 | FGFR3:TACC3\|exon 17:exon 11\|InFrame\|119\|2\|chr4:1808661:+/chr4:1741429:+ |
| 39 | EGFR | EGFR:SEPT14\|exon 24:exon 10\|InFrame\|5\|0\|chr7:55268106:+/chr7:55863785:- |
| 40 | MET | ST7:MET\|exon 3:exon 2\|NotInFrame\|264\|5\|chr7:116759774:+/chr7:116339125:+ |
| 41 | PDGFRB | SLC26A2:PDGFRB\|exon 1:exon 2\|NotInFrame\|3\|0\|chr5:149340542:+/chr5:149516616:- |
| 42 | FGFR3 | FGFR3:TACC3\|exon 17:exon 11\|InFrame\|30\|0\|chr4:1808661:+/chr4:1741429:+ |
| 43 | MET | PTPRZ1:MET\|exon 1:exon 2\|NotInFrame\|18\|3\|chr7:121513611:+/chr7:116339125:+ |
| 44 | ROS1 | GOPC:ROS1\|exon 8:exon 35\|InFrame\|6\|0\|chr6:117888017:-/chr6:117642557:- |
| 45 | EGFR | EGFR:SEPT14\|exon 24:exon 10\|InFrame\|11\|0\|chr7:55268106:+/chr7:55863785:- |
| 46 | NTRK2 | TLE4:NTRK2\|exon 8:exon 16\|InFrame\|151\|14\|chr9:82267709:+/chr9:87482158:+ |
| 47 | EGFR | EGFR:PSPH\|exon 24:exon 8\|InFrame\|8\|0\|chr7:55268106:+/chr7:56079562:- |
| 48 | MET | PTPRZ1:MET\|exon 11:exon 4\|InFrame\|321\|24\|chr7:121644714:+/chr7:116380004:+ |
| 49 | PDGFRA | CTDSP2:PDGFRA\|exon 1:exon 9\|InFrame\|10\|0\|chr12:58240155:-/chr4:55138561:+ |
| 50 | EWSR1 | EWSR1:PATZ1\|exon 10:exon 1\|InFrame\|19\|0\|chr22:29686437:+/chr22:31740641:- |
| 51 | FGFR3 | FGFR3:TACC3\|exon 17:exon 11\|InFrame\|117\|1\|chr4:1808661:+/chr4:1741429:+ |
| 52 | EGFR | EGFR:SEPT14\|exon 24:exon 10\|InFrame\|16\|3\|chr7:55268106:+/chr7:55863785:- |
| 53 | EGFR | EGFR:SEPT14\|exon 24:exon 10\|InFrame\|5\|0\|chr7:55268106:+/chr7:55863785:- |
| 54 | ABL1 | BCR:ABL1\|exon 1:exon 2\|InFrame\|4\|0\|chr22:23524426:+/chr9:133729451:+ |
| 55 | FGFR3 | FGFR3:TACC3\|exon 17:exon 8\|InFrame\|4\|0\|chr4:1808661:+/chr4:1737458:+ |
| 56 | PIK3CA | ZMAT3:PIK3CA\|exon 3:exon 2\|NotInFrame\|13\|0\|chr3:178785271:-/chr3:178916538:+ |
| 57 | EGFR | EGFR:SEPT14\|exon 24:exon 10\|InFrame\|2\|2\|chr7:55268106:+/chr7:55863785:- |
| 58 | MET | ST7:MET\|exon 1:exon 2\|NotInFrame\|15\|2\|chr7:116593745:+/chr7:116339125:+ |
| 59 | MET | PTPRZ1:MET\|exon 3:exon 2\|NotInFrame\|4\|0\|chr7:121608184:+/chr7:116339125:+ |
| 60 | FGFR3 | FGFR3:TACC3\|exon 17:exon 8\|InFrame\|71\|0\|chr4:1808661:+/chr4:1737458:+ |
| 61 | FGFR3 | FGFR3:TACC3\|exon 17:exon 11\|InFrame\|8\|1\|chr4:1808661:+/chr4:1741429:+ |
| 62 | FGFR3 | FGFR3:TACC3\|exon 17:exon 11\|InFrame\|5\|0\|chr4:1808661:+/chr4:1741429:+ |
| 63 | EGFR | EGFR:PSPH\|exon 24:exon 8\|InFrame\|937\|11\|chr7:55268106:+/chr7:56079562:- |
| 64 | MET | PTPRZ1:MET\|exon 1:exon 2\|NotInFrame\|7\|0\|chr7:121513611:+/chr7:116339125:+ |
| 65 | FGFR3 | FGFR3:TACC3\|exon 18:exon 13\|InFrame\|109\|2\|chr4:1808977:+/chr4:1742600:+ |
| 66 | FGFR3 | FGFR3:CGNL1\|exon 17:exon 10\|InFrame\|149\|1\|chr4:1808661:+/chr15:57808978:+ |
| 67 | FGFR3 | FGFR3:TACC3\|exon 17:exon 10\|InFrame\|27\|0\|chr4:1808661:+/chr4:1739325:+ |
| 68 | MET | PTPRZ1:MET\|exon 1:exon 2\|NotInFrame\|46\|0\|chr7:121513611:+/chr7:116339125:+ |
| 69 | FGFR3 | FGFR3:TACC3\|exon 17:exon 11\|InFrame\|85\|0\|chr4:1808661:+/chr4:1741429:+ |
| 70 | ROS1 | CEP290:ROS1\|exon 27:exon 35\|InFrame\|79\|0\|chr12:88490665:-/chr6:117642557:- |
| 71 | FGFR3 | FGFR3:TACC3\|exon 17:exon 8\|InFrame\|106\|2\|chr4:1808661:+/chr4:1737458:+ |
| 72 | FGFR3 | FGFR3:AMBRA1\|exon 17:exon 16\|InFrame\|35\|0\|chr4:1808661:+/chr11:46439602:- |
| 73 | PDGFRA | SCFD2:PDGFRA\|exon 4:exon 2\|InFrame\|1\|1\|chr4:54139993:-/chr4:55124924:+ |
| 74 | BRAF | MYO5A:BRAF\|exon 27:exon 11\|InFrame\|5\|0\|chr15:52645802:-/chr7:140481493:- |
| 75 | EGFR | EGFR:SEPT14\|exon 24:exon 10\|InFrame\|10\|0\|chr7:55268106:+/chr7:55863785:- |
| 76 | MET | SEPT7:MET\|exon 1:exon 2\|NotInFrame\|144\|0\|chr7:35840880:+/chr7:116339125:+ |
| 77 | FGFR3 | FGFR3:PSMC3IP\|exon 17:exon 3\|InFrame\|64\|2\|chr4:1808661:+/chr17:40729320:- |
| 78 | FGFR3 | FGFR3:TACC3\|exon 18:exon 12\|InFrame\|59\|5\|chr4:1808918:+/chr4:1741709:+ |
| 79 | MET | PDIA4:MET\|exon 1:exon 2\|NotInFrame\|15\|0\|chr7:148725413:-/chr7:116339125:+ |
| 80 | MET | CAPZA2:MET\|exon 1:exon 2\|NotInFrame\|16\|0\|chr7:116502704:+/chr7:116339125:+ |
| 81 | FGFR3 | FGFR3:WEE1\|exon 17:exon 2\|InFrame\|98\|0\|chr4:1808661:+/chr11:9597435:+ |
| 82 | EGFR | VSTM2A:EGFR\|exon 1:exon 8\|InFrame\|34\|2\|chr7:54610502:+/chr7:55223523:+ |
| 83 | FGFR3 | FGFR3:TACC3\|exon 17:exon 8\|InFrame\|79\|2\|chr4:1808661:+/chr4:1737458:+ |
| 84 | NTRK2 | BCR:NTRK2\|exon 1:exon 13\|InFrame\|23\|0\|chr22:23524426:+/chr9:87359888:+ |
| 85 | EGFR | EGFR:SEPT14\|exon 24:exon 10\|InFrame\|11\|1\|chr7:55268106:+/chr7:55863785:- |
| 86 | MET | ST7:MET\|exon 1:exon 2\|NotInFrame\|16\|1\|chr7:116593745:+/chr7:116339125:+ |
| 87 | FGFR3 | FGFR3:TACC3\|exon 17:exon 10\|InFrame\|59\|0\|chr4:1808661:+/chr4:1739325:+ |
| 88 | MET | PTPRZ1:MET\|exon 1:exon 2\|NotInFrame\|64\|3\|chr7:121513611:+/chr7:116339125:+ |
| 89 | EGFR | EGFR:SEPT14\|exon 24:exon 10\|InFrame\|104\|1\|chr7:55268106:+/chr7:55863785:- |
| 90 | EGFR | LANCL2:EGFR\|exon 1:exon 7\|InFrame\|3\|0\|chr7:55433922:+/chr7:55221704:+ |
| 91 | ROS1 | GOPC:ROS1\|exon 8:exon 35\|InFrame\|17\|0\|chr6:117888017:-/chr6:117642557:- |
| 92 | EGFR | EGFR:SEPT14\|exon 24:exon 10\|InFrame\|8\|2\|chr7:55268106:+/chr7:55863785:- |
| 93 | FGFR3 | FGFR3:TACC1\|exon 17:exon 7\|InFrame\|28\|2\|chr4:1808661:+/chr8:38693680:+ |
| 94 | NTRK2 | BCR:NTRK2\|exon 1:exon 16\|InFrame\|11\|0\|chr22:23524426:+/chr9:87482158:+ |
| 95 | EGFR | EGFR:SEPT14\|exon 24:exon 10\|InFrame\|6\|0\|chr7:55268106:+/chr7:55863785:- |
| 96 | FGFR3 | FGFR3:TACC3\|exon 17:exon 11\|InFrame\|89\|0\|chr4:1808661:+/chr4:1741429:+ |
| 97 | EGFR | EGFR:MUS81\|exon 24:exon 4\|InFrame\|11\|0\|chr7:55268106:+/chr11:65629418:+ |
| 98 | FGFR3 | FGFR3:TACC3\|exon 17:exon 11\|InFrame\|75\|0\|chr4:1808661:+/chr4:1741429:+ |
| 99 | AXL | USP34:AXL\|exon 3:exon 7\|InFrame\|94\|0\|chr2:61632843:-/chr19:41743849:+ |
| 100 | EGFR | SEC61G:EGFR\|exon 2:exon 2\|InFrame\|1\|1\|chr7:54825188:-/chr7:55209979:+ |
| 101 | EGFR | EGFR:GRB2\|exon 24:exon 3\|InFrame\|3\|0\|chr7:55268106:+/chr17:73328878:- |
| 102 | FGFR3 | FGFR3:TACC3\|exon 17:exon 11\|InFrame\|157\|1\|chr4:1808661:+/chr4:1741429:+ |
| 103 | FGFR3 | FGFR3:TACC3\|exon 17:exon 11\|InFrame\|720\|7\|chr4:1808661:+/chr4:1741429:+ |
| 104 | EGFR | EGFR:SEPT14\|exon 24:exon 10\|InFrame\|12\|5\|chr7:55268106:+/chr7:55863785:- |
| 105 | PDGFRA | TMEM165:PDGFRA\|exon 1:exon 6\|InFrame\|409\|7\|chr4:56262563:+/chr4:55133456:+ |
| 106 | ROS1 | FAM184A:ROS1\|exon 14:exon 36\|InFrame\|212\|5\|chr6:119295593:-/chr6:117641193:- |
| 107 | PDGFRA | TMEM165:PDGFRA\|exon 1:exon 6\|InFrame\|260\|11\|chr4:56262563:+/chr4:55133456:+ |
| 108 | FGFR3 | FGFR3:TACC3\|exon 17:exon 11\|InFrame\|46\|0\|chr4:1808661:+/chr4:1741429:+ |
| 109 | FGFR3 | FGFR3:TACC3\|exon 17:exon 11\|InFrame\|164\|0\|chr4:1808661:+/chr4:1741429:+ |
| 110 | MET | PTPRZ1:MET\|exon 1:exon 2\|NotInFrame\|53\|3\|chr7:121513611:+/chr7:116339125:+ |
| 111 | EGFR | EGFR:SEPT14\|exon 24:exon 10\|InFrame\|6\|0\|chr7:55268106:+/chr7:55863785:- |
| 112 | ROS1 | GOPC:ROS1\|exon 8:exon 35\|InFrame\|25\|0\|chr6:117888017:-/chr6:117642557:- |
| 113 | MET | PTPRZ1:MET\|exon 1:exon 2\|NotInFrame\|39\|2\|chr7:121513611:+/chr7:116339125:+ |
| 114 | MET | HIPK2:MET\|exon 1:exon 2\|NotInFrame\|7\|0\|chr7:139477404:-/chr7:116339125:+ |
| 115 | FGFR3 | FGFR3:TACC3\|exon 17:exon 11\|InFrame\|121\|0\|chr4:1808661:+/chr4:1741429:+ |
| 116 | MET | PTPRZ1:MET\|exon 2:exon 2\|NotInFrame\|12\|0\|chr7:121568275:+/chr7:116339125:+ |
| 117 | MET | PTPRZ1:MET\|exon 1:exon 2\|NotInFrame\|180\|16\|chr7:121513611:+/chr7:116339125:+ |
| 118 | MET | ST7:MET\|exon 1:exon 2\|NotInFrame\|4\|0\|chr7:116593745:+/chr7:116339125:+ |
| 119 | FGFR3 | FGFR3:TACC3\|exon 17:exon 11\|InFrame\|622\|7\|chr4:1808661:+/chr4:1741429:+ |
| 120 | PRKCA | SPAG9:PRKCA\|exon 8:exon 6\|InFrame\|10\|0\|chr17:49091593:-/chr17:64683229:+ |
| 121 | FGFR3 | FGFR3:TACC3\|exon 17:exon 8\|InFrame\|36\|0\|chr4:1808661:+/chr4:1737458:+ |
| 122 | FGFR3 | FGFR3:CGNL1\|exon 17:exon 10\|InFrame\|33\|1\|chr4:1808661:+/chr15:57808978:+ |
| 123 | NTRK2 | STRN:NTRK2\|exon 9:exon 12\|InFrame\|10\|0\|chr2:37111075:-/chr9:87356807:+ |
| 124 | NTRK2 | SPECC1L:NTRK2\|exon 5:exon 13\|InFrame\|120\|0\|chr22:24720395:+/chr9:87359888:+ |
| 125 | FGFR3 | FGFR3:TACC3\|exon 17:exon 11\|InFrame\|132\|2\|chr4:1808661:+/chr4:1741429:+ |
| 126 | FGFR3 | FGFR3:TACC3\|exon 17:exon 11\|InFrame\|33\|0\|chr4:1808661:+/chr4:1741429:+ |
| 127 | ETV1 | ISPD:ETV1\|exon 1:exon 9\|InFrame\|4\|0\|chr7:16460691:-/chr7:13971374:- |
| 128 | MET | ST7:MET\|exon 1:exon 2\|NotInFrame\|23\|9\|chr7:116593745:+/chr7:116339125:+ |
| 129 | EGFR | EGFR:SEPT14\|exon 24:exon 10\|InFrame\|2\|1\|chr7:55268106:+/chr7:55863785:- |
| 130 | EGFR | EGFR:SEPT14\|exon 24:exon 10\|InFrame\|4\|1\|chr7:55268106:+/chr7:55863785:- |
| 131 | MET | PTPRZ1:MET\|exon 8:exon 2\|NotInFrame\|386\|6\|chr7:121624171:+/chr7:116339125:+ |
| 132 | FGFR3 | FGFR3:TACC3\|exon 17:exon 8\|InFrame\|55\|1\|chr4:1808661:+/chr4:1737458:+ |
| 133 | FGFR3 | FGFR3:TACC3\|exon 17:exon 10\|InFrame\|1096\|19\|chr4:1808661:+/chr4:1739325:+ |
| 134 | FGFR3 | FGFR3:RALBP1\|exon 17:exon 8\|InFrame\|81\|3\|chr4:1808661:+/chr18:9533333:+ |
| 135 | FGFR3 | FGFR3:TACC3\|exon 17:exon 11\|InFrame\|435\|1\|chr4:1808661:+/chr4:1741429:+ |
| 136 | EGFR | EGFR:VSTM2A\|exon 27:exon 5\|NotInFrame\|7\|0\|chr7:55270318:+/chr7:54636824:+ |
| 137 | FGFR3 | FGFR3:TACC3\|exon 17:exon 8\|InFrame\|636\|9\|chr4:1808661:+/chr4:1737458:+ |
| 138 | PDGFRA | GPI:PDGFRA\|exon 4:exon 2\|InFrame\|9\|0\|chr19:34859607:+/chr4:55124924:+ |
| 139 | FGFR3 | FGFR3:TACC3\|exon 17:exon 11\|InFrame\|612\|3\|chr4:1808661:+/chr4:1741429:+ |
| 140 | EGFR | EGFR:PSPH\|exon 24:exon 8\|InFrame\|130\|0\|chr7:55268106:+/chr7:56079562:- |
| 141 | FGFR3 | FGFR3:TACC3\|exon 17:exon 8\|InFrame\|30\|0\|chr4:1808661:+/chr4:1737458:+ |
| 142 | BRAF | KIAA1549:BRAF\|exon 10:exon 9\|InFrame\|14\|2\|chr7:138579088:-/chr7:140487384:- |
| 143 | FGFR3 | FGFR3:TACC3\|exon 17:exon 8\|InFrame\|294\|3\|chr4:1808661:+/chr4:1737458:+ |
| 144 | EGFR | CTDSP2:EGFR\|exon 1:exon 8\|InFrame\|63\|0\|chr12:58240155:-/chr7:55223523:+ |
| 145 | TERT | SEMA5A:TERT\|exon 5:exon 2\|InFrame\|11\|1\|chr5:9318484:-/chr5:1294781:- |
| 146 | FGFR3 | FGFR3:TACC3\|exon 17:exon 8\|InFrame\|270\|0\|chr4:1808661:+/chr4:1737458:+ |
| 147 | EGFR | SEC61G:EGFR\|exon 3:exon 12\|InFrame\|88\|2\|chr7:54823472:-/chr7:55227832:+ |
| 148 | BRAF | KIAA1549:BRAF\|exon 10:exon 9\|InFrame\|5\|0\|chr7:138579088:-/chr7:140487384:- |
| 149 | ALK | ATIC:ALK\|exon 7:exon 20\|InFrame\|3\|0\|chr2:216191701:+/chr2:29446394:- |
| 150 | RET | CCDC6:RET\|exon 2:exon 12\|InFrame\|23\|0\|chr10:61612311:-/chr10:43612032:+ |
| 151 | EGFR | PTPRZ1:EGFR\|exon 1:exon 2\|InFrame\|74\|6\|chr7:121513611:+/chr7:55209979:+ |
| 152 | NTRK2 | KCTD8:NTRK2\|exon 1:exon 16\|InFrame\|31\|0\|chr4:44449580:-/chr9:87482158:+ |
| 153 | FGFR3 | FGFR3:TACC3\|exon 17:exon 11\|InFrame\|196\|4\|chr4:1808661:+/chr4:1741429:+ |
| 154 | FGFR3 | FGFR3:TACC3\|exon 17:exon 11\|InFrame\|161\|3\|chr4:1808661:+/chr4:1741429:+ |
| 155 | PDGFRA | TMEM165:PDGFRA\|exon 1:exon 6\|InFrame\|434\|7\|chr4:56262563:+/chr4:55133456:+ |
| 156 | FGFR3 | FGFR3:TACC3\|exon 17:exon 11\|InFrame\|185\|0\|chr4:1808661:+/chr4:1741429:+ |
| 157 | EGFR | SEC61G:EGFR\|exon 1:exon 2\|NotInFrame\|42\|0\|chr7:54826851:-/chr7:55209979:+ |
| 158 | FGFR3 | FGFR3:TACC3\|exon 17:exon 10\|InFrame\|10\|0\|chr4:1808661:+/chr4:1739325:+ |
| 159 | FGFR3 | FGFR3:TACC3\|exon 17:exon 11\|InFrame\|130\|0\|chr4:1808661:+/chr4:1741429:+ |
| 160 | FGFR3 | FGFR3:TACC3\|exon 17:exon 6\|InFrame\|143\|0\|chr4:1808661:+/chr4:1732899:+ |
| 161 | MET | PTPRZ1:MET\|exon 8:exon 2\|NotInFrame\|873\|31\|chr7:121624171:+/chr7:116339125:+ |
| 162 | FGFR3 | FGFR3:PAPSS1\|exon 17:exon 6\|InFrame\|315\|4\|chr4:1808661:+/chr4:108581228:- |
| 163 | FGFR3 | FGFR3:TACC3\|exon 17:exon 11\|InFrame\|449\|0\|chr4:1808661:+/chr4:1741429:+ |
| 164 | MET | PTPRZ1:MET\|exon 1:exon 2\|NotInFrame\|26\|0\|chr7:121513611:+/chr7:116339125:+ |
| 165 | NTRK2 | BCR:NTRK2\|exon 1:exon 17\|InFrame\|66\|2\|chr22:23524426:+/chr9:87549077:+ |
| 166 | FGFR3 | FGFR3:TACC3\|exon 18:exon 11\|InFrame\|658\|11\|chr4:1808947:+/chr4:1741498:+ |
| 167 | ALK | YIPF4:ALK\|exon 4:exon 14\|InFrame\|49\|0\|chr2:32523380:+/chr2:29456562:- |
| 168 | BRAF | CSPG5:BRAF\|exon 3:exon 8\|InFrame\|38\|0\|chr3:47614176:-/chr7:140494267:- |
| 169 | PDGFRA | TMEM165:PDGFRA\|exon 1:exon 6\|InFrame\|163\|1\|chr4:56262563:+/chr4:55133456:+ |
| 170 | FGFR3 | FGFR3:TACC3\|exon 17:exon 11\|InFrame\|881\|2\|chr4:1808661:+/chr4:1741429:+ |
| 171 | RELA | C11orf95:RELA\|exon 2:exon 2\|InFrame\|11\|0\|chr11:63533279:-/chr11:65429676:- |
| 172 | FGFR3 | FGFR3:TACC3\|exon 17:exon 8\|InFrame\|663\|3\|chr4:1808661:+/chr4:1737458:+ |
| 173 | MET | CAPZA2:MET\|exon 2:exon 2\|NotInFrame\|18\|0\|chr7:116528244:+/chr7:116339125:+ |
| 174 | MET | CAPZA2:MET\|exon 1:exon 2\|NotInFrame\|15\|0\|chr7:116502704:+/chr7:116339125:+ |
| 175 | MET | ST7:MET\|exon 1:exon 2\|NotInFrame\|34\|0\|chr7:116660683:+/chr7:116339125:+ |
| 176 | EGFR | MAD1L1:EGFR\|exon 2:exon 6\|InFrame\|9\|0\|chr7:1976323:-/chr7:55220239:+ |
| 177 | FGFR3 | FGFR3:TACC3\|exon 17:exon 8\|InFrame\|680\|25\|chr4:1808661:+/chr4:1737458:+ |
| 178 | EGFR | SEC61G:EGFR\|exon 1:exon 15\|NotInFrame\|4\|0\|chr7:54826851:-/chr7:55232973:+ |
| 179 | EGFR | LANCL2:EGFR\|exon 2:exon 2\|InFrame\|182\|4\|chr7:55459603:+/chr7:55209979:+ |
| 180 | MET | CAPZA2:MET\|exon 3:exon 2\|NotInFrame\|4\|1\|chr7:116533099:+/chr7:116339125:+ |
| 181 | PRKCB | NOMO1:PRKCB\|exon 20:exon 6\|InFrame\|59\|0\|chr16:14969279:+/chr16:24104112:+ |
| 182 | MET | ST7:MET\|exon 1:exon 2\|NotInFrame\|73\|4\|chr7:116593745:+/chr7:116339125:+ |
| 183 | FGFR3 | FGFR3:TACC3\|exon 17:exon 10\|InFrame\|67\|0\|chr4:1808661:+/chr4:1739325:+ |
| 184 | FGFR3 | FGFR3:TACC3\|exon 17:exon 8\|InFrame\|41\|0\|chr4:1808661:+/chr4:1737458:+ |
| 185 | ALK | CLIP2:ALK\|exon 11:exon 20\|InFrame\|4\|0\|chr7:73795193:+/chr2:29446394:- |
| 186 | ROS1 | CEP85L:ROS1\|exon 13:exon 35\|InFrame\|4\|0\|chr6:118790235:-/chr6:117642557:- |
| 187 | PDGFRA | TMEM165:PDGFRA\|exon 1:exon 6\|InFrame\|367\|5\|chr4:56262563:+/chr4:55133456:+ |
| 188 | BRAF | NRF1:BRAF\|exon 5:exon 9\|InFrame\|15\|0\|chr7:129330386:+/chr7:140487384:- |
| 189 | FGFR3 | FGFR3:TACC3\|exon 17:exon 10\|InFrame\|248\|0\|chr4:1808661:+/chr4:1739325:+ |
| 190 | EGFR | SEC61G:EGFR\|exon 1:exon 2\|NotInFrame\|9\|0\|chr7:54826851:-/chr7:55209979:+ |
| 191 | EGFR | SEC61G:EGFR\|exon 1:exon 2\|NotInFrame\|9\|0\|chr7:54826851:-/chr7:55209979:+ |
| 192 | FGFR3 | FGFR3:CEP131\|exon 17:exon 21\|InFrame\|7\|0\|chr4:1808661:+/chr17:79166192:- |
| 193 | EGFR | SEC61G:EGFR\|exon 1:exon 8\|NotInFrame\|41\|0\|chr7:54826851:-/chr7:55223523:+ |
| 194 | FGFR3 | FGFR3:WDCP\|exon 17:exon 2\|NotInFrame\|40\|0\|chr4:1808661:+/chr2:24262382:- |
| 195 | MET | PTPRZ1:MET\|exon 1:exon 2\|NotInFrame\|6\|0\|chr7:121513611:+/chr7:116339125:+ |
| 196 | FGFR3 | FGFR3:STK4\|exon 17:exon 11\|InFrame\|7\|0\|chr4:1808661:+/chr20:43703659:+ |
| 197 | MET | ST7:MET\|exon 1:exon 2\|NotInFrame\|4\|0\|chr7:116593745:+/chr7:116339125:+ |
| 198 | MET | CAPZA2:MET\|exon 1:exon 2\|NotInFrame\|109\|0\|chr7:116502704:+/chr7:116339125:+ |
| 199 | FGFR3 | FGFR3:CGNL1\|exon 17:exon 16\|InFrame\|29\|0\|chr4:1808661:+/chr15:57835894:+ |
| 200 | FGFR3 | FGFR3:TACC3\|exon 17:exon 11\|InFrame\|196\|1\|chr4:1808661:+/chr4:1741429:+ |
| 201 | FGFR3 | FGFR3:TACC3\|exon 18:exon 8\|InFrame\|599\|9\|chr4:1808856:+/chr4:1737496:+ |
| 202 | EGFR | EGFR:AMPH\|exon 24:exon 12\|InFrame\|41\|2\|chr7:55268106:+/chr7:38475988:- |
| 203 | MET | ST7:MET\|exon 1:exon 2\|NotInFrame\|4\|0\|chr7:116660683:+/chr7:116339125:+ |
| 204 | EGFR | ITCH:EGFR\|exon 2:exon 14\|NotInFrame\|622\|0\|chr20:32957276:+/chr7:55231426:+ |
| 205 | EGFR | EGFR:SEPT14\|exon 24:exon 10\|InFrame\|9\|0\|chr7:55268106:+/chr7:55863785:- |
| 206 | PDGFRA | TMEM165:PDGFRA\|exon 1:exon 6\|InFrame\|618\|31\|chr4:56262563:+/chr4:55133456:+ |
| 207 | EGFR | VOPP1:EGFR\|exon 5:exon 2\|InFrame\|1\|1\|chr7:55559975:-/chr7:55209979:+ |
| 208 | FGFR3 | FGFR3:UBXN2A\|exon 17:exon 4\|InFrame\|44\|0\|chr4:1808661:+/chr2:24199839:+ |
| 209 | FGFR3 | FGFR3:TACC3\|exon 17:exon 11\|InFrame\|1936\|38\|chr4:1808661:+/chr4:1741429:+ |
| 210 | FGFR3 | FGFR3:TACC3\|exon 17:exon 11\|InFrame\|125\|1\|chr4:1808661:+/chr4:1741429:+ |
| 211 | MET | ST7:MET\|exon 2:exon 3\|InFrame\|7\|0\|chr7:116739898:+/chr7:116371722:+ |
| 212 | FGFR3 | FGFR3:TACC3\|exon 17:exon 11\|InFrame\|94\|0\|chr4:1808661:+/chr4:1741429:+ |
| 213 | FGFR3 | FGFR3:TACC3\|exon 17:exon 11\|InFrame\|688\|8\|chr4:1808661:+/chr4:1741429:+ |
| 214 | MET | CAPZA2:MET\|exon 1:exon 2\|NotInFrame\|10\|0\|chr7:116502704:+/chr7:116339125:+ |
| 215 | FGFR3 | FGFR3:TACC3\|exon 17:exon 11\|InFrame\|87\|0\|chr4:1808661:+/chr4:1741429:+ |
| 216 | FGFR3 | FGFR3:TACC3\|exon 17:exon 11\|InFrame\|732\|7\|chr4:1808661:+/chr4:1741429:+ |
| 217 | MET | PTPRZ1:MET\|exon 1:exon 2\|NotInFrame\|189\|8\|chr7:121513611:+/chr7:116339125:+ |
| 218 | FGFR3 | FGFR3:TACC3\|exon 17:exon 11\|InFrame\|775\|10\|chr4:1808661:+/chr4:1741429:+ |
| 219 | FGFR3 | FGFR3:TACC3\|exon 17:exon 10\|InFrame\|16\|0\|chr4:1808661:+/chr4:1739325:+ |
| 220 | ROS1 | CLTA:ROS1\|exon 4:exon 33\|InFrame\|13\|0\|chr9:36204176:+/chr6:117647577:- |
| 221 | NUTM1 | RAP1GDS1:NUTM1\|exon 2:exon 3\|InFrame\|20\|0\|chr4:99214666:+/chr15:34640170:+ |
| 222 | FGFR3 | FGFR3:TACC3\|exon 17:exon 11\|InFrame\|147\|0\|chr4:1808661:+/chr4:1741429:+ |
| 223 | FGFR3 | FGFR3:TACC3\|exon 17:intron 6\|NotInFrame\|42\|0\|chr4:1808646:+/chr4:1736966:+ |
| 224 | FGFR3 | FGFR3:TACC3\|exon 17:exon 11\|InFrame\|101\|0\|chr4:1808661:+/chr4:1741429:+ |
| 225 | FGFR3 | FGFR3:COG4\|exon 17:exon 13\|InFrame\|283\|13\|chr4:1808661:+/chr16:70524295:- |
| 226 | FGFR3 | FGFR3:TACC3\|exon 17:exon 8\|InFrame\|94\|3\|chr4:1808661:+/chr4:1737458:+ |
| 227 | FGFR3 | FGFR3:TACC3\|exon 17:exon 11\|InFrame\|425\|0\|chr4:1808661:+/chr4:1741429:+ |
| 228 | PDGFRA | TMEM165:PDGFRA\|exon 1:exon 6\|InFrame\|332\|4\|chr4:56262563:+/chr4:55133456:+ |
| 229 | FGFR3 | FGFR3:TACC3\|exon 17:exon 8\|InFrame\|128\|0\|chr4:1808661:+/chr4:1737458:+ |
| 230 | FGFR3 | FGFR3:TACC3\|exon 17:exon 11\|InFrame\|208\|3\|chr4:1808661:+/chr4:1741429:+ |
| 231 | FGFR3 | FGFR3:TACC3\|exon 17:exon 8\|InFrame\|78\|1\|chr4:1808661:+/chr4:1737458:+ |
| 232 | FGFR3 | FGFR3:ZEB1\|exon 17:exon 6\|InFrame\|16\|0\|chr4:1808661:+/chr10:31803531:+ |
| 233 | NTRK2 | FIP1L1:NTRK2\|exon 12:exon 17\|InFrame\|33\|1\|chr4:54294350:+/chr9:87549077:+ |
| 234 | FGFR3 | FGFR3:TACC3\|exon 17:exon 8\|InFrame\|87\|2\|chr4:1808661:+/chr4:1737458:+ |
| 235 | NTRK2 | KCTD8:NTRK2\|exon 1:exon 16\|InFrame\|71\|3\|chr4:44449580:-/chr9:87482158:+ |
| 236 | MET | PTPRZ1:MET\|exon 1:exon 2\|NotInFrame\|81\|1\|chr7:121513611:+/chr7:116339125:+ |
| 237 | FGFR3 | FGFR3:TACC3\|exon 17:exon 11\|InFrame\|145\|0\|chr4:1808661:+/chr4:1741429:+ |
| 238 | MET | PTPRZ1:MET\|exon 2:exon 2\|NotInFrame\|29\|1\|chr7:121568275:+/chr7:116339125:+ |
| 239 | MET | CTTNBP2:MET\|exon 4:exon 15\|InFrame\|54\|0\|chr7:117431182:-/chr7:116414935:+ |
| 240 | PDGFRA | TMEM165:PDGFRA\|exon 1:exon 6\|InFrame\|5\|0\|chr4:56262563:+/chr4:55133456:+ |
| 241 | EGFR | SEC61G:EGFR\|exon 1:exon 2\|NotInFrame\|6\|0\|chr7:54826851:-/chr7:55209979:+ |
| 242 | NTRK3 | EIF2AK4:NTRK3\|exon 2:exon 4\|InFrame\|18\|0\|chr15:40231818:+/chr15:88727530:- |
| 243 | FGFR3 | FGFR3:TACC3\|exon 17:exon 11\|InFrame\|69\|0\|chr4:1808661:+/chr4:1741429:+ |
| 244 | PIK3CA | EIF4A2:PIK3CA\|exon 1:exon 2\|NotInFrame\|3\|0\|chr3:186501428:+/chr3:178916538:+ |
| 245 | ROS1 | GOPC:ROS1\|exon 7:exon 35\|InFrame\|16\|0\|chr6:117888017:-/chr6:117642557:- |
| 246 | FGFR3 | FGFR3:TACC3\|exon 17:exon 8\|InFrame\|96\|0\|chr4:1808661:+/chr4:1737458:+ |
| 247 | MET | PTPRZ1:MET\|exon 1:exon 2\|NotInFrame\|107\|1\|chr7:121513611:+/chr7:116339125:+ |
| 248 | EGFR | SEC61G:EGFR\|exon 1:exon 15\|NotInFrame\|34\|0\|chr7:54826851:-/chr7:55232973:+ |
| 249 | FGFR3 | FGFR3:TACC3\|exon 17:exon 11\|InFrame\|853\|3\|chr4:1808661:+/chr4:1741429:+ |
| 250 | FGFR3 | FGFR3:TACC3\|exon 17:exon 11\|InFrame\|629\|2\|chr4:1808661:+/chr4:1741429:+ |
| 251 | EGFR | SEC61G:EGFR\|exon 1:exon 15\|NotInFrame\|10\|0\|chr7:54826851:-/chr7:55232973:+ |
| 252 | PDGFRA | USP46:PDGFRA\|exon 1:exon 2\|InFrame\|80\|2\|chr4:53525282:-/chr4:55124924:+ |
| 253 | FGFR3 | FGFR3:TACC3\|exon 17:exon 10\|InFrame\|4\|0\|chr4:1808661:+/chr4:1739325:+ |
| 254 | RAF1 | QKI:RAF1\|exon 2:exon 8\|InFrame\|30\|0\|chr6:163876453:+/chr3:12641914:- |
| 255 | FGFR3 | FGFR3:TACC3\|exon 17:exon 11\|InFrame\|90\|0\|chr4:1808661:+/chr4:1741429:+ |
| 256 | FGFR3 | FGFR3:TACC3\|exon 17:exon 10\|InFrame\|111\|1\|chr4:1808661:+/chr4:1739325:+ |
| 257 | EGFR | SEC61G:EGFR\|exon 3:exon 14\|InFrame\|368\|7\|chr7:54823472:-/chr7:55231426:+ |
| 258 | FGFR3 | FGFR3:TACC3\|exon 17:exon 8\|InFrame\|7\|0\|chr4:1808661:+/chr4:1737458:+ |
| 259 | FGFR3 | FGFR3:TACC3\|exon 17:exon 10\|InFrame\|39\|0\|chr4:1808661:+/chr4:1739325:+ |
| 260 | EGFR | EGFR:SEPT14\|exon 27:exon 8\|InFrame\|19\|1\|chr7:55270318:+/chr7:55874951:- |
| 261 | FGFR3 | FGFR3:TACC3\|exon 17:exon 11\|InFrame\|191\|0\|chr4:1808661:+/chr4:1741429:+ |
| 262 | FGFR3 | FGFR3:TACC3\|exon 17:exon 11\|InFrame\|419\|0\|chr4:1808661:+/chr4:1741429:+ |
| 263 | MET | PTPRZ1:MET\|exon 1:exon 2\|NotInFrame\|19\|0\|chr7:121513611:+/chr7:116339125:+ |
| 264 | NTRK2 | BCR:NTRK2\|exon 1:exon 17\|InFrame\|33\|0\|chr22:23524426:+/chr9:87549077:+ |
| 265 | FGFR3 | FGFR3:TACC3\|exon 17:exon 10\|InFrame\|105\|2\|chr4:1808661:+/chr4:1739325:+ |
| 266 | FGFR3 | FGFR3:GLYR1\|exon 17:exon 5\|InFrame\|43\|0\|chr4:1808661:+/chr16:4873908:- |
| 267 | BCOR | CREBBP:BCOR\|exon 30:exon 5\|InFrame\|165\|0\|chr16:3778022:-/chrX:39930412:- |
| 268 | MET | PTPRZ1:MET\|exon 2:exon 2\|NotInFrame\|49\|0\|chr7:121568275:+/chr7:116339125:+ |
| 269 | PDGFRA | TMEM165:PDGFRA\|exon 1:exon 6\|InFrame\|83\|1\|chr4:56262563:+/chr4:55133456:+ |
| 270 | FGFR3 | FGFR3:GRPEL1\|exon 17:exon 3\|InFrame\|37\|0\|chr4:1808661:+/chr4:7064193:- |
| 271 | ROS1 | GOPC:ROS1\|exon 7:exon 35\|InFrame\|15\|0\|chr6:117888017:-/chr6:117642557:- |
| 272 | FGFR3 | FGFR3:TACC3\|exon 17:exon 8\|InFrame\|20\|0\|chr4:1808661:+/chr4:1737458:+ |
| 273 | EGFR | CTDSP2:EGFR\|exon 1:exon 2\|InFrame\|9\|0\|chr12:58240155:-/chr7:55209979:+ |
| 274 | FGFR3 | FGFR3:COG4\|exon 17:exon 13\|InFrame\|19\|0\|chr4:1808661:+/chr16:70524295:- |
| 275 | NTRK1 | IRF2BP2:NTRK1\|exon 2:exon 8\|InFrame\|5\|0\|chr1:234743554:-/chr1:156843425:+ |
| 276 | FGFR3 | FGFR3:TACC3\|exon 17:exon 10\|InFrame\|418\|4\|chr4:1808661:+/chr4:1739325:+ |
| 277 | EGFR | EGFR:GZMA\|exon 27:exon 2\|InFrame\|19\|0\|chr7:55270318:+/chr5:54401302:+ |
| 278 | NTRK2 | CAPRIN1:NTRK2\|exon 7:exon 16\|InFrame\|13\|0\|chr11:34101312:+/chr9:87482158:+ |
| 279 | FGFR3 | FGFR3:TACC3\|exon 17:exon 8\|InFrame\|324\|1\|chr4:1808661:+/chr4:1737458:+ |
| 280 | PRIM1 | PRIM1:GLI1\|exon 12:exon 2\|InFrame\|3\|0\|chr12:57127931:-/chr12:57858456:+ |
| 281 | FGFR3 | FGFR3:TACC3\|exon 17:exon 10\|InFrame\|8\|0\|chr4:1808661:+/chr4:1739325:+ |
| 282 | MET | ST7:MET\|exon 1:exon 2\|NotInFrame\|5\|4\|chr7:116593745:+/chr7:116339125:+ |
| 283 | NTRK2 | GKAP1:NTRK2\|exon 9:exon 16\|InFrame\|24\|0\|chr9:86363224:-/chr9:87482158:+ |
| 284 | FGFR3 | FGFR3:TACC3\|exon 17:exon 11\|InFrame\|283\|0\|chr4:1808661:+/chr4:1741429:+ |
| 285 | FGFR3 | FGFR3:TACC3\|exon 17:exon 10\|InFrame\|224\|0\|chr4:1808661:+/chr4:1739325:+ |
| 286 | FGFR3 | FGFR3:TACC3\|exon 17:exon 11\|InFrame\|488\|2\|chr4:1808661:+/chr4:1741429:+ |
| 287 | EGFR | EGFR:PALLD\|exon 27:exon 19\|InFrame\|8\|0\|chr7:55270318:+/chr4:169845406:+ |
| 288 | MET | CAPZA2:MET\|exon 1:exon 2\|NotInFrame\|5\|0\|chr7:116502704:+/chr7:116339125:+ |
| 289 | EGFR | TEK:EGFR\|exon 1:exon 2\|InFrame\|6\|0\|chr9:27109640:+/chr7:55209979:+ |
| 290 | NTRK1 | ARHGEF11:NTRK1\|exon 40:exon 12\|InFrame\|12\|0\|chr1:156906608:-/chr1:156845312:+ |
| 291 | FGFR3 | FGFR3:TACC3\|exon 17:exon 11\|InFrame\|3\|0\|chr4:1808661:+/chr4:1741429:+ |
| 292 | EGFR | JAZF1:EGFR\|exon 1:exon 8\|InFrame\|3\|0\|chr7:28220082:-/chr7:55223523:+ |
| 293 | EGFR | CALM2:EGFR\|exon 3:exon 14\|NotInFrame\|39\|0\|chr2:47397873:-/chr7:55231426:+ |
| 294 | EGFR | EGFR:LOC101927653\|exon 26:exon 8\|NotInFrame\|13\|0\|chr7:55269475:+/chr12:58960371:- |
| 295 | FGFR3 | FGFR3:TACC3\|exon 17:exon 8\|InFrame\|204\|4\|chr4:1808661:+/chr4:1737458:+ |
| 296 | FGFR3 | FGFR3:ATAD2\|exon 17:exon 2\|InFrame\|7\|0\|chr4:1808661:+/chr8:124392917:- |
| 297 | FGFR3 | FGFR3:TACC3\|exon 17:exon 10\|InFrame\|1150\|11\|chr4:1808661:+/chr4:1739325:+ |
| 298 | FGFR3 | FGFR3:TACC3\|exon 17:exon 11\|InFrame\|315\|0\|chr4:1808661:+/chr4:1741429:+ |
| 299 | FGFR3 | FGFR3:TACC3\|exon 17:exon 8\|InFrame\|146\|1\|chr4:1808661:+/chr4:1737458:+ |
| 300 | NTRK3 | RFX7:NTRK3\|exon 7:exon 14\|InFrame\|33\|3\|chr15:56393557:-/chr15:88576276:- |
| 301 | FGFR3 | FGFR3:TACC3\|exon 17:exon 11\|InFrame\|564\|0\|chr4:1808661:+/chr4:1741429:+ |
| 302 | MET | PTPRZ1:MET\|exon 2:exon 2\|NotInFrame\|56\|1\|chr7:121568275:+/chr7:116339125:+ |
| 303 | ROS1 | GOPC:ROS1\|exon 7:exon 35\|InFrame\|34\|0\|chr6:117888017:-/chr6:117642557:- |
| 304 | FGFR3 | FGFR3:TACC3\|exon 17:exon 8\|InFrame\|67\|0\|chr4:1808661:+/chr4:1737458:+ |
| 305 | MET | PTPRZ1:MET\|exon 2:exon 2\|NotInFrame\|35\|0\|chr7:121568275:+/chr7:116339125:+ |
| 306 | EGFR | SEC61G:EGFR\|exon 1:exon 5\|NotInFrame\|8\|0\|chr7:54826851:-/chr7:55218987:+ |
| 307 | EGFR | EGFR:PSPH\|exon 24:exon 8\|InFrame\|202\|0\|chr7:55268106:+/chr7:56079562:- |
| 308 | FGFR3 | FGFR3:TACC3\|exon 17:exon 11\|InFrame\|273\|0\|chr4:1808661:+/chr4:1741429:+ |
| 309 | FGFR3 | FGFR3:TACC3\|exon 17:intron 6\|NotInFrame\|598\|13\|chr4:1808655:+/chr4:1736999:+ |
| 310 | NTRK2 | SPECC1L:NTRK2\|exon 8:exon 17\|InFrame\|5\|0\|chr22:24730541:+/chr9:87549077:+ |
| 311 | FGFR3 | FGFR3:TACC3\|exon 17:exon 14\|InFrame\|4\|0\|chr4:1808661:+/chr4:1746245:+ |
| 312 | FGFR3 | FGFR3:TACC3\|exon 17:exon 11\|InFrame\|475\|1\|chr4:1808661:+/chr4:1741429:+ |
| 313 | MET | HIP1:MET\|exon 26:exon 15\|InFrame\|30\|0\|chr7:75172170:-/chr7:116414935:+ |
| 314 | BRAF | CTTNBP2:BRAF\|exon 2:exon 9\|InFrame\|9\|1\|chr7:117501263:-/chr7:140487384:- |
| 315 | FGFR3 | FGFR3:EIF2B3\|exon 17:exon 9\|InFrame\|435\|1\|chr4:1808661:+/chr1:45341367:- |
| 316 | FGR | MTF2:FGR\|exon 1:exon 2\|NotInFrame\|5\|0\|chr1:93545088:+/chr1:27951662:- |
| 317 | EGFR | TSFM:EGFR\|exon 4:exon 3\|InFrame\|9\|0\|chr12:58180945:+/chr7:55210998:+ |
| 318 | MET | PTPRZ1:MET\|exon 14:exon 2\|NotInFrame\|242\|9\|chr7:121668697:+/chr7:116339125:+ |
| 319 | CPSF6 | CPSF6:GLI1\|exon 1:exon 2\|NotInFrame\|216\|0\|chr12:69633486:+/chr12:57857448:+ |
| 320 | PDGFRA | TMEM165:PDGFRA\|exon 1:exon 6\|InFrame\|5\|0\|chr4:56262563:+/chr4:55133456:+ |
| 321 | FGFR3 | FGFR3:TACC3\|intron 17:exon 8\|NotInFrame\|77\|0\|chr4:1808695:+/chr4:1737489:+ |
| 322 | RAF1 | TMF1:RAF1\|exon 16:exon 8\|InFrame\|7\|0\|chr3:69073206:-/chr3:12641914:- |
| 323 | FGFR3 | FGFR3:TACC3\|exon 17:exon 10\|InFrame\|56\|0\|chr4:1808661:+/chr4:1739325:+ |
| 324 | FGFR3 | FGFR3:TACC3\|intron 17:intron 10\|NotInFrame\|32\|0\|chr4:1808706:+/chr4:1741396:+ |
| 325 | FGFR3 | FGFR3:TACC3\|exon 17:exon 8\|InFrame\|166\|1\|chr4:1808661:+/chr4:1737458:+ |
| 326 | FGFR3 | FGFR3:TACC3\|exon 17:exon 10\|InFrame\|35\|0\|chr4:1808661:+/chr4:1739325:+ |
| 327 | PDGFRA | TMEM165:PDGFRA\|exon 1:exon 6\|InFrame\|26\|1\|chr4:56262563:+/chr4:55133456:+ |
| 328 | MET | RAP1B:MET\|exon 1:exon 2\|NotInFrame\|82\|0\|chr12:69004823:+/chr7:116339125:+ |
| 329 | FGFR3 | FGFR3:TACC3\|exon 17:exon 8\|InFrame\|13\|0\|chr4:1808661:+/chr4:1737458:+ |
| 330 | FGFR3 | FGFR3:TACC3\|exon 17:exon 10\|InFrame\|659\|4\|chr4:1808661:+/chr4:1739325:+ |
| 331 | TERT | CCDC127:TERT\|exon 3:exon 2\|InFrame\|18\|3\|chr5:205817:-/chr5:1294781:- |
| 332 | NTRK2 | BCR:NTRK2\|exon 1:exon 17\|InFrame\|3\|0\|chr22:23524426:+/chr9:87549077:+ |
| 333 | NTRK2 | SPECC1L:NTRK2\|exon 8:exon 15\|InFrame\|8\|0\|chr22:24730541:+/chr9:87475955:+ |
| 334 | FGFR3 | FGFR3:TACC3\|exon 17:exon 8\|InFrame\|25\|0\|chr4:1808661:+/chr4:1737458:+ |
| 335 | EGFR | EGFR:AHCYL2\|exon 24:exon 2\|InFrame\|6\|0\|chr7:55268106:+/chr7:129019482:+ |
| 336 | NTRK2 | GKAP1:NTRK2\|exon 9:exon 16\|InFrame\|4\|1\|chr9:86363224:-/chr9:87482158:+ |
| 337 | FGFR3 | FGFR3:TACC3\|exon 17:exon 8\|InFrame\|66\|0\|chr4:1808661:+/chr4:1737458:+ |
| 338 | MYB | MYB:QKI\|exon 9:exon 5\|InFrame\|61\|1\|chr6:135517140:+/chr6:163983014:+ |
| 339 | FGFR3 | FGFR3:TACC3\|exon 17:exon 11\|InFrame\|48\|0\|chr4:1808661:+/chr4:1741429:+ |
| 340 | PDGFRA | TMEM165:PDGFRA\|exon 1:exon 6\|InFrame\|410\|3\|chr4:56262563:+/chr4:55133456:+ |
| 341 | FGFR3 | FGFR3:MATR3\|exon 17:exon 5\|InFrame\|57\|0\|chr4:1808661:+/chr5:138650364:+ |
| 342 | MET | ST7:MET\|exon 1:exon 2\|NotInFrame\|46\|1\|chr7:116593745:+/chr7:116339125:+ |
| 343 | NTRK2 | SPECC1L:NTRK2\|exon 10:exon 17\|InFrame\|17\|0\|chr22:24743144:+/chr9:87549077:+ |
| 344 | MET | ST7:MET\|exon 1:exon 2\|NotInFrame\|5\|0\|chr7:116593745:+/chr7:116339125:+ |
| 345 | BRAF | KIAA1549:BRAF\|exon 15:exon 9\|InFrame\|8\|0\|chr7:138552721:-/chr7:140487384:- |
| 346 | FGFR3 | FGFR3:ZC3H7B\|exon 17:exon 2\|NotInFrame\|6\|0\|chr4:1808661:+/chr22:41716659:+ |
| 347 | MET | CAPZA2:MET\|exon 1:exon 2\|NotInFrame\|9\|0\|chr7:116502704:+/chr7:116339125:+ |
| 348 | NTRK2 | BEND5:NTRK2\|exon 3:exon 16\|InFrame\|14\|0\|chr1:49224572:-/chr9:87482158:+ |
| 349 | MET | ST7:MET\|exon 1:exon 2\|NotInFrame\|3\|0\|chr7:116593745:+/chr7:116339125:+ |
| 350 | EGFR | EGFR:VSTM2A\|exon 27:exon 2\|InFrame\|3\|0\|chr7:55270318:+/chr7:54612315:+ |
| 351 | NTRK2 | BCR:NTRK2\|exon 1:exon 17\|InFrame\|29\|1\|chr22:23524426:+/chr9:87549077:+ |
| 352 | EGFR | EGFR:ADAM22\|exon 27:exon 31\|NotInFrame\|392\|7\|chr7:55270318:+/chr7:87825786:+ |
| 353 | EGFR | EGFR:PSPH\|exon 24:exon 8\|InFrame\|728\|5\|chr7:55268106:+/chr7:56079562:- |
| 354 | EGFR | EGFR:ZBTB20-AS4\|exon 24:exon 2\|NotInFrame\|685\|3\|chr7:55268106:+/chr3:114821403:+ |
| 355 | EGFR | EGFR:LOC100996654\|exon 24:exon 3\|NotInFrame\|496\|0\|chr7:55268106:+/chr7:54872359:+ |
| 356 | EGFR | SEC61G:EGFR\|exon 1:exon 2\|NotInFrame\|3\|0\|chr7:54826851:-/chr7:55209979:+ |
| 357 | FGFR3 | FGFR3:TACC3\|exon 17:exon 11\|InFrame\|127\|0\|chr4:1808661:+/chr4:1741429:+ |
| 358 | NTRK2 | BCR:NTRK2\|exon 1:exon 16\|InFrame\|7\|0\|chr22:23524426:+/chr9:87482158:+ |
| 359 | FGFR3 | FGFR3:TACC3\|exon 17:exon 11\|InFrame\|37\|0\|chr4:1808661:+/chr4:1741429:+ |
| 360 | EGFR | EGFR:LINC01446\|exon 24:exon 6\|NotInFrame\|27\|0\|chr7:55268106:+/chr7:53725141:- |
| 361 | FGFR3 | FGFR3:TDRD7\|exon 17:exon 8\|InFrame\|153\|1\|chr4:1808661:+/chr9:100232840:+ |
| 362 | NTRK2 | KANK1:NTRK2\|exon 7:exon 16\|InFrame\|16\|0\|chr9:713464:+/chr9:87482158:+ |
| 363 | EGFR | EGFR:LANCL2\|exon 26:exon 8\|InFrame\|9\|0\|chr7:55269475:+/chr7:55496068:+ |
| 364 | BRAF | KIAA1549:BRAF\|exon 15:exon 9\|InFrame\|14\|0\|chr7:138552721:-/chr7:140487384:- |
| 365 | EGFR | EGFR:SEPT14\|exon 24:exon 7\|InFrame\|722\|49\|chr7:55268106:+/chr7:55886916:- |
| 366 | FGFR3 | FGFR3:TACC3\|exon 17:exon 11\|InFrame\|419\|2\|chr4:1808661:+/chr4:1741429:+ |
| 367 | BRAF | KIAA1549:BRAF\|exon 10:exon 9\|InFrame\|10\|0\|chr7:138579088:-/chr7:140487384:- |
| 368 | FGFR3 | FGFR3:TACC3\|exon 18:exon 11\|InFrame\|755\|7\|chr4:1808875:+/chr4:1741462:+ |
| 369 | ROS1 | GOPC:ROS1\|exon 3:exon 36\|InFrame\|6\|0\|chr6:117896340:-/chr6:117641193:- |
| 370 | FGFR3 | FGFR3:TACC3\|intron 17:intron 10\|NotInFrame\|162\|0\|chr4:1808706:+/chr4:1741411:+ |
| 371 | FGFR3 | FGFR3:TACC3\|exon 18:exon 11\|InFrame\|21\|0\|chr4:1808907:+/chr4:1741437:+ |
| 372 | EGFR | EGFR:COL28A1\|exon 24:exon 16\|InFrame\|239\|7\|chr7:55268106:+/chr7:7495743:- |
| 373 | RET | CCDC6:RET\|exon 1:exon 12\|InFrame\|5\|0\|chr10:61665880:-/chr10:43612032:+ |
| 374 | NTRK2 | EML1:NTRK2\|exon 6:exon 16\|InFrame\|4\|0\|chr14:100357564:+/chr9:87482158:+ |
| 375 | FGFR3 | FGFR3:CGNL1\|exon 17:exon 14\|InFrame\|158\|7\|chr4:1808661:+/chr15:57820852:+ |
| 376 | FGFR3 | FGFR3:TACC3\|exon 17:exon 11\|InFrame\|947\|1\|chr4:1808661:+/chr4:1741429:+ |
| 377 | EGFR | EGFR:LINC01446\|exon 24:exon 6\|NotInFrame\|340\|0\|chr7:55268106:+/chr7:53725141:- |
| 378 | MET | ST7:MET\|exon 1:exon 2\|NotInFrame\|3\|0\|chr7:116593745:+/chr7:116339125:+ |
| 379 | FGFR3 | FGFR3:TACC3\|exon 17:exon 10\|InFrame\|923\|4\|chr4:1808661:+/chr4:1739325:+ |
| 380 | CTDSP2 | CTDSP2:GLI1\|exon 1:exon 2\|InFrame\|3\|0\|chr12:58240155:-/chr12:57858456:+ |
| 381 | NTRK3 | RFX7:NTRK3\|exon 7:exon 14\|InFrame\|25\|3\|chr15:56393557:-/chr15:88576276:- |
| 382 | RAF1 | MKRN2:RAF1\|exon 6:exon 8\|InFrame\|178\|6\|chr3:12623451:+/chr3:12641914:- |
| 383 | MET | AUTS2:MET\|exon 6:exon 2\|NotInFrame\|50\|1\|chr7:70163606:+/chr7:116339125:+ |
| 384 | EGFR | SEC61G:EGFR\|exon 1:exon 15\|NotInFrame\|66\|0\|chr7:54826851:-/chr7:55232973:+ |
| 385 | MET | PTPRZ1:MET\|exon 1:exon 2\|NotInFrame\|12\|0\|chr7:121513611:+/chr7:116339125:+ |
| 386 | EGFR | VOPP1:EGFR\|exon 5:exon 12\|InFrame\|3\|1\|chr7:55559975:-/chr7:55227852:+ |
| 387 | MET | PRKAR2B:MET\|exon 1:exon 15\|InFrame\|3\|0\|chr7:106685659:+/chr7:116414935:+ |
| 388 | FGFR3 | FGFR3:TACC3\|exon 17:exon 11\|InFrame\|459\|0\|chr4:1808661:+/chr4:1741429:+ |
| 389 | FGFR3 | FGFR3:TACC3\|exon 17:exon 10\|InFrame\|139\|4\|chr4:1808661:+/chr4:1739325:+ |
| 390 | FGFR3 | FGFR3:TACC3\|exon 17:exon 10\|InFrame\|355\|1\|chr4:1808661:+/chr4:1739325:+ |
| 391 | CPSF6 | CPSF6:GLI1\|exon 1:exon 2\|NotInFrame\|7\|0\|chr12:69633486:+/chr12:57857448:+ |
| 392 | MET | CAPZA2:MET\|exon 1:exon 2\|NotInFrame\|3\|0\|chr7:116502704:+/chr7:116339125:+ |
| 393 | EGFR | UBE2L3:EGFR\|exon 1:exon 15\|InFrame\|5\|0\|chr22:21922060:+/chr7:55232973:+ |
| 394 | NTRK2 | BCR:NTRK2\|exon 1:exon 16\|InFrame\|45\|2\|chr22:23524426:+/chr9:87482158:+ |
| 395 | MYBL1 | MYBL1:MMP16\|exon 9:exon 2\|InFrame\|11\|0\|chr8:67492368:-/chr8:89209535:- |
| 396 | MET | PTPRZ1:MET\|exon 1:exon 2\|NotInFrame\|293\|11\|chr7:121513611:+/chr7:116339125:+ |
| 397 | FGFR3 | FGFR3:TACC3\|exon 17:exon 14\|InFrame\|3\|0\|chr4:1808661:+/chr4:1746245:+ |
| 398 | EGFR | SEC61G:EGFR\|exon 2:exon 2\|InFrame\|3\|0\|chr7:54825188:-/chr7:55209979:+ |
| 399 | MET | GTF2I:MET\|exon 6:exon 15\|InFrame\|17\|1\|chr7:74114964:+/chr7:116414935:+ |
| 400 | EGFR | NUP62:EGFR\|exon 2:exon 15\|NotInFrame\|16\|0\|chr19:50430951:-/chr7:55232973:+ |
| 401 | MET | PTPRZ1:MET\|exon 17:exon 2\|NotInFrame\|300\|2\|chr7:121674432:+/chr7:116339125:+ |
| 402 | MET | ST7:MET\|exon 1:exon 2\|NotInFrame\|19\|1\|chr7:116660683:+/chr7:116339125:+ |
| 403 | MET | ST7:MET\|exon 1:exon 2\|NotInFrame\|10\|1\|chr7:116593745:+/chr7:116339125:+ |
| 403 | MET | CAPZA2:MET\|exon 1:exon 2\|NotInFrame\|6\|1\|chr7:116502704:+/chr7:116339125:+ |
| 404 | MET | CAPZA2:MET\|exon 1:exon 2\|NotInFrame\|76\|0\|chr7:116502704:+/chr7:116339125:+ |
| 404 | MET | CAPZA2:MET\|exon 1:exon 3\|InFrame\|4\|0\|chr7:116502704:+/chr7:116371722:+ |
| 405 | EGFR | EGFR:XRCC5\|exon 24:exon 20\|InFrame\|155\|0\|chr7:55268106:+/chr2:217069045:+ |
| 405 | EGFR | IGFBP2:EGFR\|exon 1:exon 8\|InFrame\|136\|1\|chr2:217498688:+/chr7:55223523:+ |
| 406 | MET | CAPZA2:MET\|exon 1:exon 2\|NotInFrame\|46\|0\|chr7:116502704:+/chr7:116339125:+ |
| 406 | MET | CAPZA2:MET\|exon 1:exon 3\|InFrame\|4\|0\|chr7:116502704:+/chr7:116371722:+ |
| 407 | MET | CAPZA2:MET\|exon 1:exon 2\|NotInFrame\|473\|2\|chr7:116502704:+/chr7:116339125:+ |
| 407 | MET | CAPZA2:MET\|exon 1:exon 3\|InFrame\|29\|2\|chr7:116502704:+/chr7:116371722:+ |
| 408 | MET | ST7:MET\|exon 1:exon 2\|NotInFrame\|19\|0\|chr7:116660683:+/chr7:116339125:+ |
| 408 | MET | PTPRZ1:MET\|exon 1:exon 2\|NotInFrame\|15\|2\|chr7:121513611:+/chr7:116339125:+ |
| 409 | MET | ST7:MET\|exon 1:exon 2\|NotInFrame\|4\|0\|chr7:116660683:+/chr7:116339125:+ |
| 409 | PDGFRA | CHIC2:PDGFRA\|exon 3:exon 2\|InFrame\|31\|0\|chr4:54915122:-/chr4:55124924:+ |
| 409 | PDGFRA | CTDSP2:PDGFRA\|exon 1:exon 11\|InFrame\|13\|1\|chr12:58240155:-/chr4:55140698:+ |
| 410 | RET | NCOA4:RET\|exon 9:exon 12\|InFrame\|3\|0\|chr10:51586411:+/chr10:43612032:+ |
| 410 | ROS1 | NHSL1:ROS1\|exon 1:exon 32\|InFrame\|23\|1\|chr6:138892847:-/chr6:117650609:- |
| 410 | ROS1 | TBC1D32:ROS1\|exon 25:exon 32\|InFrame\|22\|0\|chr6:121452773:-/chr6:117650609:- |
| 411 | R3HDM2 | R3HDM2:GLI1\|exon 2:exon 2\|NotInFrame\|78\|0\|chr12:57789532:-/chr12:57857448:+ |
| 411 | CTDSP2 | CTDSP2:GLI1\|exon 1:exon 2\|InFrame\|21\|0\|chr12:58240155:-/chr12:57858456:+ |
| 412 | PDGFRA | SCFD2:PDGFRA\|exon 4:exon 6\|InFrame\|519\|8\|chr4:54139993:-/chr4:55133456:+ |
| 412 | PDGFRA | SCFD2:PDGFRA\|exon 4:exon 2\|InFrame\|19\|9\|chr4:54139993:-/chr4:55124924:+ |
| 413 | MET | ST7:MET\|exon 1:exon 2\|NotInFrame\|225\|14\|chr7:116660683:+/chr7:116339125:+ |
| 413 | MET | ST7:MET\|exon 1:exon 2\|NotInFrame\|90\|10\|chr7:116593745:+/chr7:116339125:+ |
| 413 | MET | CAPZA2:MET\|exon 2:exon 2\|NotInFrame\|5\|0\|chr7:116528244:+/chr7:116339125:+ |
| 414 | RELA | C11orf95:RELA\|exon 3:exon 2\|InFrame\|72\|0\|chr11:63532340:-/chr11:65429676:- |
| 414 | RELA | C11orf95:RELA\|exon 3:exon 3\|InFrame\|9\|0\|chr11:63532340:-/chr11:65429559:- |
| 414 | RELA | C11orf95:RELA\|exon 2:exon 2\|InFrame\|4\|0\|chr11:63533279:-/chr11:65429676:- |
| 415 | FGFR3 | FGFR3:TACC3\|exon 17:exon 11\|InFrame\|199\|0\|chr4:1808661:+/chr4:1741429:+ |
| 415 | MET | PTPRZ1:MET\|exon 1:exon 2\|NotInFrame\|1\|1\|chr7:121513611:+/chr7:116339125:+ |
| 415 | NTRK2 | BCR:NTRK2\|exon 1:exon 17\|InFrame\|99\|2\|chr22:23524426:+/chr9:87549077:+ |
| 416 | FGFR2 | FGFR2:ATAD2\|exon 17:exon 2\|InFrame\|45\|0\|chr10:123243212:-/chr8:124392917:- |
| 416 | FGFR3 | FGFR3:TACC3\|exon 18:intron 8\|NotInFrame\|6\|0\|chr4:1808918:+/chr4:1738728:+ |
| 417 | FGFR3 | FGFR3:TACC3\|exon 17:exon 11\|InFrame\|308\|0\|chr4:1808661:+/chr4:1741429:+ |
| 417 | IKZF2 | IKZF2:ERBB4\|exon 4:exon 2\|InFrame\|7\|0\|chr2:214012432:-/chr2:212989628:- |
| 418 | FGFR3 | FGFR3:TACC3\|exon 17:exon 11\|InFrame\|47\|0\|chr4:1808661:+/chr4:1741429:+ |
| 418 | ROS1 | TDP2:ROS1\|exon 2:exon 36\|InFrame\|1\|1\|chr6:24666754:-/chr6:117641193:- |
| 419 | EGFR | SEC61G:EGFR\|exon 2:exon 8\|InFrame\|35\|0\|chr7:54825188:-/chr7:55223523:+ |
| 419 | EGFR | SEC61G:EGFR\|exon 1:exon 8\|NotInFrame\|9\|0\|chr7:54826851:-/chr7:55223523:+ |
| 420 | MET | ST7:MET\|exon 1:exon 2\|NotInFrame\|5\|0\|chr7:116660683:+/chr7:116339125:+ |
| 420 | MET | ST7:MET\|exon 1:exon 2\|NotInFrame\|3\|0\|chr7:116593745:+/chr7:116339125:+ |
| 421 | MET | CAPZA2:MET\|exon 2:exon 2\|NotInFrame\|52\|0\|chr7:116528244:+/chr7:116339125:+ |
| 421 | PDGFRA | TMEM165:PDGFRA\|exon 1:exon 6\|InFrame\|5\|0\|chr4:56262563:+/chr4:55133456:+ |
| 422 | MET | CAPZA2:MET\|exon 2:exon 2\|NotInFrame\|43\|0\|chr7:116528244:+/chr7:116339125:+ |
| 422 | MET | ST7:MET\|exon 1:exon 2\|NotInFrame\|26\|2\|chr7:116593745:+/chr7:116339125:+ |
| 423 | MET | CAPZA2:MET\|exon 5:exon 13\|InFrame\|151\|0\|chr7:116544437:+/chr7:116411552:+ |
| 423 | MET | CAPZA2:MET\|exon 1:exon 2\|NotInFrame\|18\|0\|chr7:116502704:+/chr7:116339125:+ |
| 424 | EGFR | EGFR:SEPT14\|exon 26:exon 10\|InFrame\|674\|4\|chr7:55269475:+/chr7:55863785:- |
| 424 | EGFR | EGFR:SEPT14\|exon 27:exon 8\|InFrame\|10\|4\|chr7:55270318:+/chr7:55874951:- |
| 425 | EGFR | SEC61G:EGFR\|exon 3:exon 14\|InFrame\|1222\|18\|chr7:54823472:-/chr7:55231426:+ |
| 425 | EGFR | EGFR:PSPH\|exon 24:exon 8\|InFrame\|14\|0\|chr7:55268106:+/chr7:56079562:- |
| 426 | NTRK2 | SPECC1L:NTRK2\|exon 8:exon 17\|InFrame\|5\|0\|chr22:24730541:+/chr9:87549077:+ |
| 426 | R3HDM2 | R3HDM2:GLI1\|exon 2:exon 2\|NotInFrame\|9\|0\|chr12:57789532:-/chr12:57857448:+ |
| 427 | PDGFRA | NMU:PDGFRA\|exon 2:exon 6\|InFrame\|16\|1\|chr4:56496569:-/chr4:55133456:+ |
| 427 | PDGFRA | TMEM165:PDGFRA\|exon 1:exon 6\|InFrame\|8\|1\|chr4:56262563:+/chr4:55133456:+ |
| 428 | NTRK2 | CRLF3:NTRK2\|exon 7:exon 15\|InFrame\|15\|0\|chr17:29112937:-/chr9:87475955:+ |
| 428 | NTRK2 | CRLF3:NTRK2\|exon 7:exon 16\|InFrame\|9\|0\|chr17:29112937:-/chr9:87482158:+ |
