# Supplementary material for: Fusion transcriptome landscape in glioblastoma: Incidence and therapeutic implications
Source: Neurooncol Adv. 2025 Nov 13;8(1):vdaf238. doi: 10.1093/noajnl/vdaf238 (PMC12831933; doi:10.1093/noajnl/vdaf238)
Supplement: vdaf238_Supplementary_Data [file vdaf238_supplementary_data.zip › Supplementary table 2.docx]

| Index | Fusion | Details | TKI | TKI After TMZ? | Overall Survival | *Survival Status | Age | Gender | Race | Ethnicity |
| --- | --- | --- | --- | --- | --- | --- | --- | --- | --- | --- |
| 185 | ALK | CLIP2:ALK\|exon 11:exon 20\|InFrame\|4\|0\|chr7:73795193:+/chr2:29446394:- | Alectinib | Yes | 25.97 | 1 | 57 | Male | White | Not Hispanic or Latino |
| 319 | CPSF6 | CPSF6:GLI1\|exon 1:exon 2\|NotInFrame\|216\|0\|chr12:69633486:+/chr12:57857448:+ | Nintedanib | No | 14.53 | 1 | 75 | Male | White | Not Hispanic or Latino |
| 52 | EGFR | EGFR:SEPT14\|exon 24:exon 10\|InFrame\|16\|3\|chr7:55268106:+/chr7:55863785:- | Imatinib | No | 27.60 | 0 | 59 | Male | - | - |
| 237 | FGFR3 | FGFR3:TACC3\|exon 17:exon 11\|InFrame\|145\|0\|chr4:1808661:+/chr4:1741429:+ | Infigratinib | Yes | 8.40 | 0 | 55 | Female | White | Not Hispanic or Latino |
| 159 | FGFR3 | FGFR3:TACC3\|exon 17:exon 11\|InFrame\|130\|0\|chr4:1808661:+/chr4:1741429:+ | Erdafitinib | No TMZ on Record | 27.27 | 0 | 55 | Male | - | - |
| 375 | FGFR3 | FGFR3:CGNL1\|exon 17:exon 14\|InFrame\|158\|7\|chr4:1808661:+/chr15:57820852:+ | Erdafitinib | Yes | 8.47 | 1 | 56 | Male | Unknown | Not Hispanic or Latino |
| 30 | FGFR3 | FGFR3:TACC3\|exon 17:exon 14\|InFrame\|2\|1\|chr4:1808661:+/chr4:1746245:+ | Erdafitinib | Yes | 20.40 | 0 | 58 | Male | White | Not Hispanic or Latino |
| 297 | FGFR3 | FGFR3:TACC3\|exon 17:exon 10\|InFrame\|1150\|11\|chr4:1808661:+/chr4:1739325:+ | Erdafitinib | Yes | 9.97 | 0 | 59 | Male | - | - |
| 210 | FGFR3 | FGFR3:TACC3\|exon 17:exon 11\|InFrame\|125\|1\|chr4:1808661:+/chr4:1741429:+ | Infigratinib | Yes | 17.87 | 0 | 64 | Female | White | Not Hispanic or Latino |
| 208 | FGFR3 | FGFR3:UBXN2A\|exon 17:exon 4\|InFrame\|44\|0\|chr4:1808661:+/chr2:24199839:+ | Erdafitinib, Infigratinib | Yes | 9.60 | 0 | 70 | Male | White | Not Hispanic or Latino |
| 209 | FGFR3 | FGFR3:TACC3\|exon 17:exon 11\|InFrame\|1936\|38\|chr4:1808661:+/chr4:1741429:+ | Erdafitinib | No TMZ on Record | 23.30 | 1 | 41 | Female | - | - |
| 222 | FGFR3 | FGFR3:TACC3\|exon 17:exon 11\|InFrame\|147\|0\|chr4:1808661:+/chr4:1741429:+ | Erdafitinib | No TMZ on Record | 22.07 | 1 | 70 | Male | White | Not Hispanic or Latino |
| 272 | FGFR3 | FGFR3:TACC3\|exon 17:exon 8\|InFrame\|20\|0\|chr4:1808661:+/chr4:1737458:+ | Erdafitinib | Yes | 19.80 | 1 | 72 | Female | White | Not Hispanic or Latino |
| 62 | FGFR3 | FGFR3:TACC3\|exon 17:exon 11\|InFrame\|5\|0\|chr4:1808661:+/chr4:1741429:+ | Erdafitinib | Yes | 5.43 | 0 | 43 | Male | Asian or Pacific Islander | Not Hispanic or Latino |
| 135 | FGFR3 | FGFR3:TACC3\|exon 17:exon 11\|InFrame\|435\|1\|chr4:1808661:+/chr4:1741429:+ | Infigratinib | Yes | 23.43 | 0 | 53 | Female | White | Not Hispanic or Latino |
| 35 | FGFR3 | FGFR3:PAPSS1\|exon 17:exon 6\|InFrame\|36\|1\|chr4:1808661:+/chr4:108581228:- | Regorafenib | Yes | 41.97 | 0 | 57 | Male | White | Unknown |
| 55 | FGFR3 | FGFR3:TACC3\|exon 17:exon 8\|InFrame\|4\|0\|chr4:1808661:+/chr4:1737458:+ | Olaparib | Yes | 28.17 | 0 | 58 | Female | Unknown | Not Hispanic or Latino |
| 71 | FGFR3 | FGFR3:TACC3\|exon 17:exon 8\|InFrame\|106\|2\|chr4:1808661:+/chr4:1737458:+ | Regorafenib | Yes | 11.70 | 0 | 59 | Male | Other | Hispanic or Latino |
| 249 | FGFR3 | FGFR3:TACC3\|exon 17:exon 11\|InFrame\|853\|3\|chr4:1808661:+/chr4:1741429:+ | Vismodegib | Yes | 15.93 | 0 | 61 | Female | White | Not Hispanic or Latino |
| 415 | FGFR3 | FGFR3:TACC3\|exon 17:exon 11\|InFrame\|199\|0\|chr4:1808661:+/chr4:1741429:+ | Larotrectinib | Yes | 7.23 | 0 | 62 | Female | White | Not Hispanic or Latino |
| 404 | MET | CAPZA2:MET\|exon 1:exon 2\|NotInFrame\|76\|0\|chr7:116502704:+/chr7:116339125:+ | Abemaciclib, Crizotinib, Lorlatinib | Yes | 16.17 | 0 | 64 | Male | Unknown | Not Hispanic or Latino |
| 305 | MET | PTPRZ1:MET\|exon 2:exon 2\|NotInFrame\|35\|0\|chr7:121568275:+/chr7:116339125:+ | Cabozantinib | Yes | 13.93 | 1 | 66 | Male | White | Not Hispanic or Latino |
| 175 | MET | ST7:MET\|exon 1:exon 2\|NotInFrame\|34\|0\|chr7:116660683:+/chr7:116339125:+ | Crizotinib | Yes | 20.33 | 0 | 68 | Female | White | Not Hispanic or Latino |
| 37 | MET | CAPZA2:MET\|exon 2:exon 2\|NotInFrame\|36\|0\|chr7:116528244:+/chr7:116339125:+ | Crizotinib | Yes | 3.53 | 0 | 71 | Male | Asian or Pacific Islander | Unknown |
| 80 | MET | CAPZA2:MET\|exon 1:exon 2\|NotInFrame\|16\|0\|chr7:116502704:+/chr7:116339125:+ | Crizotinib | Yes | 12.43 | 0 | 72 | Male | White | Not Hispanic or Latino |
| 6 | MET | TNRC6B:MET\|exon 1:exon 2\|NotInFrame\|24\|2\|chr22:40574144:+/chr7:116339125:+ | Sirolimus | Yes | 2.63 | 0 | 60 | Male | White | Not Hispanic or Latino |
| 275 | NTRK1 | IRF2BP2:NTRK1\|exon 2:exon 8\|InFrame\|5\|0\|chr1:234743554:-/chr1:156843425:+ | Larotrectinib | Yes | 9.47 | 0 | 32 | Male | - | - |
| 333 | NTRK2 | SPECC1L:NTRK2\|exon 8:exon 15\|InFrame\|8\|0\|chr22:24730541:+/chr9:87475955:+ | Entrectinib | No TMZ on Record | 11.77 | 1 | 60 | Male | Asian or Pacific Islander | Unknown |
| 165 | NTRK2 | BCR:NTRK2\|exon 1:exon 17\|InFrame\|66\|2\|chr22:23524426:+/chr9:87549077:+ | Larotrectinib | Yes | 16.33 | 0 | 61 | Female | White | Not Hispanic or Latino |
| 336 | NTRK2 | GKAP1:NTRK2\|exon 9:exon 16\|InFrame\|4\|1\|chr9:86363224:-/chr9:87482158:+ | Larotrectinib | Yes | 11.57 | 1 | 66 | Male | White | Not Hispanic or Latino |
| 343 | NTRK2 | SPECC1L:NTRK2\|exon 10:exon 17\|InFrame\|17\|0\|chr22:24743144:+/chr9:87549077:+ | Larotrectinib | Yes | 15.07 | 1 | 68 | Female | White | Not Hispanic or Latino |
| 123 | NTRK2 | STRN:NTRK2\|exon 9:exon 12\|InFrame\|10\|0\|chr2:37111075:-/chr9:87356807:+ | Entrectinib | Yes | 34.30 | 0 | 72 | Male | White | Not Hispanic or Latino |
| 358 | NTRK2 | BCR:NTRK2\|exon 1:exon 16\|InFrame\|7\|0\|chr22:23524426:+/chr9:87482158:+ | Entrectinib | No TMZ on Record | 9.77 | 1 | 75 | Female | Black or African American | Not Hispanic or Latino |
| 428 | NTRK2 | CRLF3:NTRK2\|exon 7:exon 15\|InFrame\|15\|0\|chr17:29112937:-/chr9:87475955:+ | Larotrectinib | No TMZ on Record | 9.07 | 1 | 53 | Female | White | Not Hispanic or Latino |
| 351 | NTRK2 | BCR:NTRK2\|exon 1:exon 17\|InFrame\|29\|1\|chr22:23524426:+/chr9:87549077:+ | Selinexor | No TMZ on Record | 2.33 | 0 | 57 | Male | - | - |
| 169 | PDGFRA | TMEM165:PDGFRA\|exon 1:exon 6\|InFrame\|163\|1\|chr4:56262563:+/chr4:55133456:+ | Regorafenib | Yes | 18.60 | 0 | 28 | Male | White | Not Hispanic or Latino |
| 187 | PDGFRA | TMEM165:PDGFRA\|exon 1:exon 6\|InFrame\|367\|5\|chr4:56262563:+/chr4:55133456:+ | Avapritinib | Yes | 20.87 | 0 | 26 | Male | Asian or Pacific Islander | Not Hispanic or Latino |
| 49 | PDGFRA | CTDSP2:PDGFRA\|exon 1:exon 9\|InFrame\|10\|0\|chr12:58240155:-/chr4:55138561:+ | Abemaciclib | Yes | 14.30 | 0 | 50 | Male | Other | Unknown |
| 244 | PIK3CA | EIF4A2:PIK3CA\|exon 1:exon 2\|NotInFrame\|3\|0\|chr3:186501428:+/chr3:178916538:+ | Aflibercept | No | 14.97 | 0 | 84 | Male | White | Not Hispanic or Latino |
| 322 | RAF1 | TMF1:RAF1\|exon 16:exon 8\|InFrame\|7\|0\|chr3:69073206:-/chr3:12641914:- | Regorafenib | Yes | 13.93 | 1 | 61 | Female | White | Not Hispanic or Latino |
| 410 | RET, ROS1 | NCOA4:RET\|exon 9:exon 12\|InFrame\|3\|0\|chr10:51586411:+/chr10:43612032:+ | Entrectinib, Pralsetinib, Selpercatinib | Yes | 13.43 | 0 | 61 | Female | Unknown | Not Hispanic or Latino |
| 410 | RET, ROS1 | TBC1D32:ROS1\|exon 25:exon 32\|InFrame\|22\|0\|chr6:121452773:-/chr6:117650609:- | Entrectinib, Pralsetinib, Selpercatinib | Yes | 13.43 | 0 | 61 | Female | Unknown | Not Hispanic or Latino |

*1:Censored, 0:Event

Supplementary table 2: Clinical characteristics of the patients who received TKIs
